# Attribute latencies causally shape intertemporal decisions

**DOI:** 10.1038/s41467-024-46657-2

**Published:** 2024-04-05

**Authors:** Fadong Chen, Jiehui Zheng, Lei Wang, Ian Krajbich

**Affiliations:** 1 School of Management, Zhejiang University, Hangzhou, 310058 China; 2 Neuromanagement Laboratory, Zhejiang University, Hangzhou, 310058 China; 3 The State Key Laboratory of Brain-Machine Intelligence, Zhejiang University, Hangzhou, 310058 China; 4 Alibaba Business School, Hangzhou Normal University, Hangzhou, 311121 China; 5 Department of Psychology, University of California Los Angeles, Los Angeles, CA USA

**Keywords:** Human behaviour, Economics

## Abstract

Intertemporal choices – decisions that play out over time – pervade our life. Thus, how people make intertemporal choices is a fundamental question. Here, we investigate the role of attribute latency (the time between when people start to process different attributes) in shaping intertemporal preferences using five experiments with choices between smaller-sooner and larger-later rewards. In the first experiment, we identify attribute latencies using mouse-trajectories and find that they predict individual differences in choices, response times, and changes across time constraints. In the other four experiments we test the causal link from attribute latencies to choice, staggering the display of the attributes. This changes attribute latencies and intertemporal preferences. Displaying the amount information first makes people more patient, while displaying time information first does the opposite. These findings highlight the importance of intra-choice dynamics in shaping intertemporal choices and suggest that manipulating attribute latency may be a useful technique for nudging.

## Abstract (web summary)

People have different latencies in processing amount and time attributes when making intertemporal choices. Here, the authors test the causal effect of these latencies on choice by altering the onset of amount and time information, which alters people’s patience.

## Introduction

Intertemporal choices that involve tradeoffs between outcomes available at different times are ubiquitous in our everyday life. These tradeoffs play an important role in many personal decisions and policy questions, such as saving, education, exercise, health care, nutrition, and so forth. Thus, understanding how people form intertemporal preferences and act on those preferences are fundamental issues in economics, psychology, and other social sciences, as well as to designing public policies or nudge interventions^1–9^. Economists typically analyze intertemporal choices and design public policies using utility models that assume discount rates on delayed rewards^10–14^. If we take these utility models as descriptions of how people actually decide, then we assume a static model of the decision process where the amount and time attributes are integrated within each option and the best option is selected, all instantaneously.

On the other hand, there have been efforts to understand the dynamics of intertemporal choice, with both single- and dual-process models. In the dual-process studies, intertemporal choices are described as an interaction between automatic and deliberative processes. Some have argued that people automatically favor immediate rewards^15–19^, while others have argued the opposite^20,21^. Time manipulations are often used to induce people to rely more on the intuitive process or the deliberative process when making decisions. For instance, past work has argued that time pressure encourages automatic responses, while time delay encourages more deliberative responses^22^. However, other work has instead argued that time pressure increases reliance on prior information (predispositions) while time delay increases the evaluation of the choice options^23^. More generally, it is not clear whether changes in choice behavior due to time constraints are the result of a shift between automatic and deliberative processes, or due to other components (e.g., processing biases or attentional priorities) of the choice process^24–26^.

Single-process studies have attempted to examine the dynamics underlying intertemporal choice using sequential sampling models (SSMs), which account for both choice and response time (RT) data. These studies have argued that intertemporal choice involves attribute-wise processes in which amount and time are evaluated and compared separately^5,27–31^. Hence, this perspective sees intertemporal choices as resulting from the combination of amount comparisons and time comparisons. Moreover, using computational modeling, this line of studies has shown that people have predispositions and evaluation biases when making intertemporal decisions^24^, and participants are heterogeneous in the relative speed of processing the amount and time attributes, where the difference between the onset times of the amount and time attributes is referred to as attribute latency^3,28,32^.

Prior work has analyzed attribute latencies using computational modeling based on choice and RT^3,28,32^ but an alternative approach would be to directly measure participants’ attribute latencies with mouse-trajectory data. Mouse-tracking—measuring the computer-mouse movements made by participants while making decisions—is a real-time technique to more directly tap into the processes underlying choices^33–37^. The rich temporal data offered by mouse-tracking allow us to test nuanced models regarding how decisions unfold^33^. Recent research has highlighted the usefulness of mouse trajectories in multi-attribute choice, in particular for inferring when attributes enter the decision process^38–44^.

In this work, we use five separate experiments to investigate the role that attribute latency plays in shaping intertemporal preferences and whether we can manipulate participants’ attribute latencies to causally change their intertemporal choices. The first experiment (Study 1) combines mouse-tracking with a standard intertemporal choice paradigm where participants chose between smaller-sooner (SS) and larger-later (LL) monetary rewards with and without time constraints. We estimate participants’ attribute latencies, i.e., the differences between the onset time of processing amount and time information (time-onset lag, TOL), using the mouse-trajectory data only. We find that the mouse-trajectory-derived time-onset lag (MTTOL) predicts individual differences in choices, RTs, and the behavioral changes across time constraints. We validate these results using an independent computational modeling analysis in which the attribute latency is estimated using choice and RT data. The mouse-trajectory data in Study 1 not only allows us to identify an independent measure of attribute latency, but also shows how the attribute latency changes across time constraints. We find that time constraints do not affect attribute latencies in the same way. Time pressure pushes attribute latencies in opposite directions depending on whether they are positive or negative. This aligns with how behavior changes under time pressure, i.e., patient people become more patient under time pressure while impatient people become less patient under time pressure. Given the correlation between attribute latencies and behavior, we also test the causal pathway from attribute latencies to intertemporal choices using four experiments (Studies 2-5). Specifically, we manipulate attribute latencies by altering the onset of the amount and time information. We find that displaying the amount information first makes people process the amount information earlier than the time information, while displaying the time information first has the opposite effect. More importantly, this causally changes choices, i.e., people chose more patient (impatient) options when we display the amount (time) attribute first.

## Results

### Intertemporal choice task

**Fig. 1:**
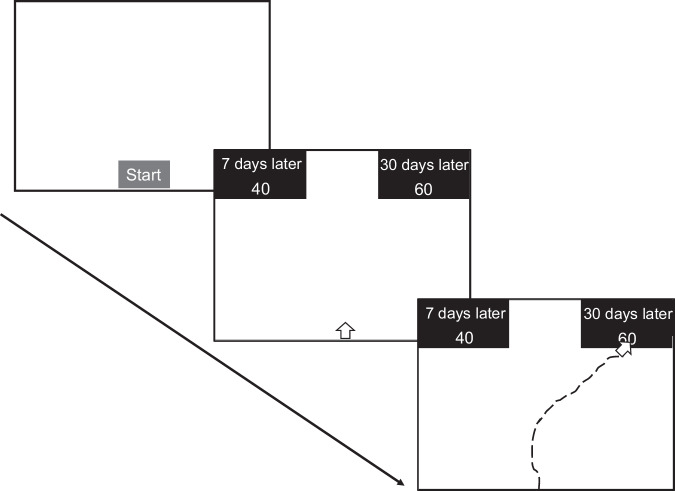
An example and the timeline of the intertemporal choice task in Study 1. At the beginning of each trial, participants clicked the “Start” button in the middle bottom of the screen, and then the decision problem appeared. In this example, the choice was between getting a reward of 40 in 7 days and getting a reward of 60 in 30 days. Participants made decisions by moving their mouse to their favored option and clicking the black area of that option. The text was enlarged for display purposes.

Study 1 consisted of 300 intertemporal choice trials (the same 300 trials were used in Studies 2–5) with and without time constraints. In each trial, participants decided between two options, one with a large reward but delivered at a later time (LL or patient option), the other with a small reward but delivered at a sooner time (SS or impatient option) (Fig. 1). That is, making decisions in each trial required deciding whether the additional money offered at the later date made it worth the extra delay.

We divided the 300 trials into four blocks. The first and the last blocks were time-free blocks (with 100 trials in each). The other two blocks in between were time-pressure and time-delay blocks (with 50 trials in each). Participants had to make each decision within 2 s in the time-pressure block, and they had to make each decision after the decision problem had been displayed for 10 s in the time-delay block. In the two time-free blocks, participants could take as long as they wanted to make each decision. The order of time-pressure and time-delay blocks was counterbalanced across participants. The spatial positions of the LL and SS options were counterbalanced across trials for each participant.

The mean RTs were 2.121 (SD = 1.230), 1.263 (SD = 0.283), and 1.192 (SD = 0.868, after the 10 s enforced delay) seconds in the time-free, time-pressure and time-delay conditions respectively. In the time-free condition, the mean percentage of choosing LL options was 58.9% (SD = 21.3%). The mean percentages of choosing LL options were 65.5% (SD = 26.2%) and 53.9% (SD = 20.6%) in the time-pressure and delay conditions, respectively. That is, for our set of trials, participants generally chose the patient option, and they became more patient under time pressure and less patient under time delay, on average (two-sided sign rank tests, pressure vs. free: *V* = 6526.5, *p* < 0.001; free vs. delay: *V* = 6222, *p* < 0.001; pressure vs. delay: *V* = 7116.5, *p* < 0.001).

### Attribute latency

Prior studies have shown that people start to process the amount and time attributes at different times when making intertemporal decisions^3,28^. Here we take the difference between the onset times of the amount and time attributes (i.e., time-onset lag, TOL) as the measure of the attribute latency. A positive TOL means that the participant starts to process the amount attribute earlier than the time attribute, while a negative TOL means that the participant starts to process the time attribute earlier than the amount attribute.

**Fig. 2:**
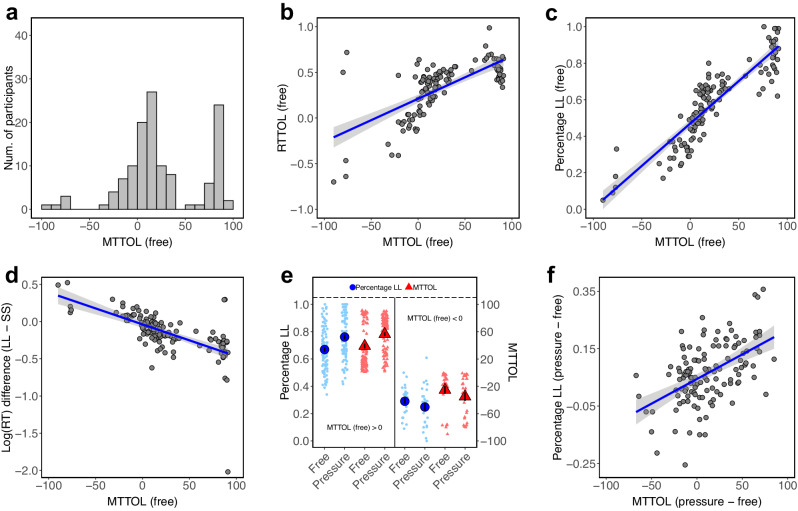
Attribute latencies determine intertemporal preferences in Study 1. **a** Distribution of the mouse-trajectory-derived time-onset lag (MTTOL) in the time-free condition. **b** Correlation between the response-time-derived time-onset lag (RTTOL) in the starting-time drift diffusion model (stDDM) and the MTTOL in the time-free condition. **c** Correlation between the MTTOL estimated using half of the time-free trials and the percentage of LL decisions in the other half of the time-free trials. **d** Correlation between the MTTOL and the log(RT) difference between LL and SS decisions in the time-free condition. **e** The MTTOL and the percentage of LL decisions across time-pressure and free conditions, separately for participants with positive and negative MTTOLs in the time-free condition. **f** Correlation between MTTOL change and behavioral change across time-pressure and free conditions. The MTTOL was computed as a fraction of the maximum RT in each condition, while the RTTOL was computed in seconds. The MTTOL in the time-free condition was estimated using half of the time-free trials, and the percentage of LL decisions in the time-free condition was computed using the other half of the time-free trials. Each dot in **b**–**d** and **f** represents one participant. The blue lines and grey shadings in **b**–**d** and **f** are the fitted linear regression lines and their standard errors. Each light blue dot (percentage of LL decision) or light red triangle (MTTOL) in **e** represents one participant. The deep blue dot and the deep red triangle in **e** represents the mean percentage of LL decisions and the mean MTTOL for a group of participants with positive or negative MTTOLs. The vertical bars in **e** represent standard errors of the mean clustered by participant.

We first estimated the attribute latency (TOL) using mouse-trajectory data only (mouse-trajectory-derived TOL, MTTOL), for each participant in each time condition (see Methods for details). Figure 2a shows the distribution of the MTTOL in the time-free condition, and Supplementary Fig. 1 shows the distributions of the MTTOL in the time-pressure and delay conditions. The mean MTTOLs were 25.41 (SD = 41.44)$,$ 37.33 (SD = 46.44), 15.24 (SD = 31.19) in the time-free, time-pressure and time-delay conditions, respectively. That is, on average, participants processed the amount attribute earlier than the time attribute (two-sided Wilcoxon signed rank test, free: *V* = 6609, *p* < 0.001; pressure: *V* = 6645.5, *p* < 0.001; delay: *V* = 5589, *p* < 0.001), and they processed the amount attribute much earlier than the time attribute in the time-pressure condition compared to the other two conditions (pressure vs. free: *V* = 9103.5, *p* = 0.044; pressure vs. delay: *V* = 11027, *p* < 0.001, free vs. delay: *V* = 9753.5, *p* = 0.002).

To validate the mouse-tracking measure of attribute latency, we estimated a starting-time drift-diffusion model (stDDM, Supplementary Fig. 4)^3,28,32,45^ at the participant level using the choice and RT data in the time-free condition (see Methods and Supplementary Note 2). The stDDM models the drift rate, which captures the rate of evidence accumulation in favor of one option over the other, as a linear function of the attribute differences and a constant. In particular, there is a delay before one of the attribute differences affects the drift rate. That is, the stDDM allows the amount and time attributes to enter into the evidence accumulation process at different times, and thus can decompose behavior into attribute latency (TOL), predisposition (starting point), evaluation bias (drift-rate constant), and subjective weights on each attribute in the evidence accumulation process (see Methods). The parameter recovery exercise revealed that earlier/later onset of evidence accumulation (TOL) can be distinguished from other components (e.g., predisposition, evaluation bias, and relative attribute weights, see Supplementary Note 2) of the process underlying intertemporal decisions.

We have to note that it is challenging to estimate the stDDM in the time-pressure and delay conditions, since it is not clear how the decision process was affected by the time constraints. For instance, time limits are usually modeled using collapsing boundaries^25,46^. However, whether boundaries collapse over time is still an active debate^47–52^. Thus, we cannot confidently estimate stDDM under time constraints and we only fit the stDDM to the data in the time-free condition.

The computational modeling results mirrored those from the mouse-trajectory analysis. On average, participants processed the amount attribute earlier than the time attribute (Mean = 0.330 s, SD = 0.288 s, two-sided Wilcox signed rank test, *V* = 7380, *p* < 0.001). In more detail, the TOLs in the stDDM (response-time-derived time-onset lag, RTTOL) were correlated with the MTTOLs across participants (Fig. 2b, two-sided Pearson correlation test, *r*(124) = 0.676, *p* < 0.001, *t* = 10.221, 95% CI = [0.569, 0.761]). The sign of the RTTOL was the same as the sign of the MTTOL for 117 out of 126 participants. For the 26 participants whose MTTOL was negative, 18 of them had a negative RTTOL (two-sided Binomial test, *p* = 0.076), and for the 99 participants whose MTTOL was positive, all of them had a positive RTTOL. This lends credence to the estimation of attribute latency based on the mouse-trajectory data.

Additionally, we estimated four other versions of the DDM (see Supplementary Note 2). The cross-validation analysis revealed that the stDDM and the standard DDM had better out-of-sample predictive performance than the other three models, while the stDDM had higher predictive performance than the standard DDM (though non-significantly). Thus, going forward we focus on comparisons between the stDDM and the standard DDM.

### Attribute latency explains individual differences in choices and response times

Next, we tested whether between-participant differences in MTTOL could explain variation in choice behavior and RTs. Prior studies have shown that attribute-wise models can sometimes better describe participants’ intertemporal choices than option-wise models^27,28,31^, while others argue that option-wise utility models work better^1,53–55^. Therefore, we opted not to fit a utility model here but used a model-free measure instead. Specifically, we computed the percentage of LL decisions for each participant and took this percentage as a measure of their intertemporal preferences (i.e., patience).

Figure 2c shows that the MTTOL identified using half of the time-free trials was correlated with the likelihood of choosing LL options in the other half of the time-free trials (two-sided Pearson correlation test, *r*(124) = 0.895, *p* < 0.001, *t* = 22.395, 95% CI = [0.854, 0.925]; also see Supplementary Fig. 6a). Moreover, MTTOLs were correlated with the percentage of LL decisions in the time-pressure and delay conditions (Supplementary Fig. 2, pressure: *r*(124) = 0.920, *p* < 0.001, *t* = 26.194, 95% CI = [0.888, 0.943]; delay: *r*(124) = 0.839, *p* < 0.001, *t* = 17.199, 95% CI = [0.779, 0.884]). That is, the attribute latency under time pressure and delay can also predict participants’ choice behavior. The OLS regressions in Supplementary Table 3 show that the MTTOL added additional power in explaining participants’ choice behavior in the time-free condition. Except the RTTOL, all the other parameters in stDDM explained 86.8% of the variance of participants’ choice behavior (model 2). Adding the MTTOL (model 3) significantly increased ${R}^{2}$ from 0.868 to 0.920 (models 2 vs 3, two-sided partial-*F* test, *F*-value = 78.184, *p* < 0.001). Moreover, the ${R}^{2}$ of the regression on standard DDM parameters (0.938) is less than that of the regression on stDDM parameters (0.945; models 1 vs. 4, two-sided partial-*F* test, *F*-value = $15.613$, *p* < 0.001).

With respect to RT, we expected MTTOL to be negatively correlated with the RT difference between LL and SS decisions in the time-free condition. The reason is that, on average, participants who process the amount information earlier (later) than the time information are quicker (slower) in making LL decisions than SS decisions. Figure 2d shows that the MTTOL was correlated with the RT difference between LL and SS decisions (two-sided Pearson correlation test, *r*(123) = −0.635, *p* < 0.001, *t* = −9.120, 95% CI = [−0.729, −0.517]; also see Supplementary Fig. 6b). The regressions in Supplementary Table 4 show that, except RTTOL, all the other parameters in stDDM explained 48.6% of the variance of the RT differences (model 2). Adding the MTTOL (model 3) significantly increased ${R}^{2}$ from 0.486 to 0.533 (models 2 vs. 3, two-sided partial-*F* test, *F*-value = 12.910, *p* < 0.001). The ${R}^{2}$ of the regression on the standard DDM parameters (0.553) is less than that of the stDDM parameters (0.573; models 1 vs. 4, two-sided partial-*F* test, *F*-value$=6.656$, *p* = 0.011). Therefore, taking attribute latency into account adds power in explaining RT differences.

### Attribute latency predicts behavioral changes across time constraints

Supplementary Fig. 7 shows an S-shaped pattern of the choice behavior across time constraints, especially in the cases of time-pressure versus free (Supplementary Fig. 7a) and time-pressure versus delay (Supplementary Fig. 7c) conditions. Participants with a high percentage of LL decisions in the time-free condition (i.e., patient participants) chose more LL options under time pressure and chose fewer LL options under time delay, while participants with a low percentage of LL decisions (i.e., impatient participants) did the opposite. That is, time constraints did not affect participants’ choice behavior in the same way, and the effects depended on the level of participants’ patience. In particular, the behavioral changes across time-pressure and delay conditions were correlated with the percentage of LL decisions in the time-free condition (Supplementary Fig. 7d, two-sided Pearson correlation test, *r*(124) = 0.367, *p* < 0.001, *t* = 4.390, 95% CI = [0.205, 0.509]). Participants who were more patient in the time-free condition became more patient when going from time delay to time pressure.

Recent studies have shown that behavioral changes across time-pressure and delay conditions can be predicted by the pre-decisional bias (captured by the starting-point parameter in the DDM), the evaluation bias (captured by the drift-rate constant), or the attentional priorities on different attributes (captured by the subjective weights)^23–25^. Here we investigate whether attribute latency can predict behavioral changes across time constraints. Supplementary Fig. 8c shows that the MTTOL in the time-free condition was correlated with the behavioral changes across time-pressure and delay conditions (two-sided Pearson correlation test, *r*(124) = 0.329, *p* < 0.001, *t* = 3.885, 95% CI = [0.164, 0.477]). The OLS regressions in Supplementary Table 5 show that taking attribute latency into account can better explain the directions and magnitudes of behavioral changes across time conditions (see Supplementary Note 4 for more details).

### Attribute latency changes are in line with the behavioral changes across time constraints

In the time-delay condition, participants could only start to move the mouse after the options had been displayed for 10 s. Moreover, participants’ decisions were fastest (on average, after the 10 s enforced delay) in the time-delay condition (mean = 1.192 s). Thus, it is clear that participants began to make their choices before moving the mouse. Therefore, here we only analyze how the MTTOL changed across time-free and pressure conditions.

Participants whose MTTOL was positive in the time-free condition processed the amount attribute much earlier than the time attribute under time pressure (Fig. 2e, two-sided Wilcoxon signed rank test, *V* = 1148, *p* < 0.001), while participants whose MTTOL was negative processed the time attribute earlier than the amount attribute under time pressure (Fig. 2e, *V* = 231.5, *p* = 0.065). This shows a similar S-shaped pattern as the behavioral change across time-pressure and free conditions (Supplementary Fig. 9). That is, time pressure sped up the processing of one attribute over the other. More importantly, the MTTOL changes were aligned with the behavioral changes for the two groups of participants (Fig. 2e). Compared to the time-free condition, participants with positive MTTOLs chose more LL options (two-sided Wilcoxon signed rank test, *V* = 4481.5, *p* < 0.001), while participants with negative MTTOLs chose fewer LL options (*V* = 89, *p* = 0.027) in the time-pressure condition. The MTTOL change was correlated with the behavioral change across participants (Fig. 2f, two-sided Pearson correlation test, *r*(124) = 0.515, *p* < 0.001, *t* = 6.693, 95% CI = [0.374, 0.633]).

We also tested whether MTTOL mediated the effect of time constraint on the choice behavior. Both time condition and MTTOL entered into a linear regression model predicting choice behavior. The mediation analysis revealed that the effect of time constraint was significantly reduced (from *β* = 0.065, *p* = 0.032 to *β* = 0.006, *p* = 0.632), and that MTTOL was a significant predictor of choice behavior (*β* = 0.005, *p* < 0.001). Using a bootstrapping method (with 10, 000 iterations), we tested the significance of the indirect effect of time constraint on choice behavior through MTTOL. The 95% confidence interval for the indirect effect did not include zero (0.003, 0.110), indicating significant mediation (Supplementary Fig. 10).

### Manipulating intertemporal choice behavior via attribute latency

The results above show that attribute latency (TOL) is malleable. To test the causal link from attribute latencies to intertemporal choices, we ran four experiments (Studies 2–5) with two different manipulations and with/out mouse-tracking. Studies 2 and 3 are purely behavioral studies. In these two studies, we manipulated the order in which we displayed the amount and time information and tested whether this altered the likelihood that participants made a patient choice. Compared to Study 1, we changed the time-pressure and time-delay blocks into amount-first and time-first blocks in Study 2. In the amount-first block, the amount attribute for each option was first displayed for 3 s, followed by the time attribute (along with the amount information). In the time-first block, the time attribute for each option was first displayed for 3 s, followed by the amount attribute (along with the time information). Participants were free to make decisions at any time. In Study 3, we first displayed one attribute for 1.5 s, before the other attribute appeared. Moreover, participants could not make decisions in the first 1.5 s in Study 3. Otherwise, the two studies (2 and 3) were identical. Studies 4 and 5 were replications of Studies 2 and 3 respectively, with mouse-tracking.

The central idea here is that displaying one attribute first may induce participants to start processing this attribute first. We expected that, compared to the control condition, the TOL would increase in the amount-first condition and decrease in the time-first condition. Based on the results of Study 1, this would make participants more patient (choose LL options more) in the amount-first condition and less patient (choose LL options less) in the time-first condition.

**Fig. 3:**
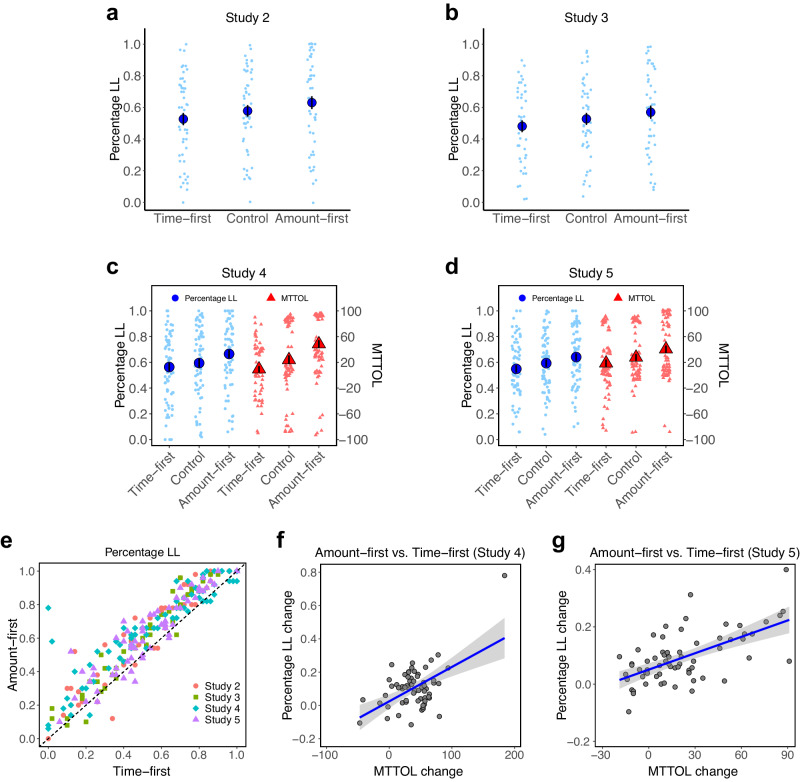
Attribute latencies causally shape intertemporal choices in Studies 2–5. **a**, **b** The percentage of LL decisions across manipulation conditions in Studies 2 and 3; **c**, **d** The percentage of LL decisions (blue dots) and the mouse-trajectory-derived time-onset lag (MTTOL, red triangles) across manipulation conditions in Studies 4 and 5; **e** the percentage of LL decisions across time-first and amount-first conditions for each participant in Studies 2–5. **f**, **g** Correlation between the MTTOL change and the behavioral change across time-first and amount-first conditions in Studies 4 and 5. Each light blue dot or light red triangle in **a**–**d** represents one participant. The deep blue dot in **a**–**d** represents the mean percentage of LL decisions and the deep red triangle in **c**, **d** represents the mean MTTOL. The vertical bars in **a**–**d** represent the standard errors of the mean clustered by participant. Each data point in **e**–**g** represents one participant. The black dashed line in **e** is the identity line. The blue solid lines and gray shadings in **f**, **g** are the fitted linear regression lines and their standard errors.

In line with our predictions, participants chose more LL options in the amount-first condition (Study 2: mean = 0.630, SD = 0.285; Study 3: mean = 0.570; SD = 0.274; Study 4: mean = 0.666, SD = 0.255; Study 5: mean = 0.639, SD = 0.224) than the control condition (Study 2: mean = 0.578, SD = 0.258; Study 3: mean = 0.528; SD = 0.249; Study 4: mean = 0.593, SD = 0.273; Study 5: mean = 0.592, SD = 0.215; two-sided Wilcoxon signed rank tests, Study 2: *V* = 151, *p* < 0.001; Study 3: *V* = 222.5, *p* = 0.007; Study 4: *V* = 180, *p* < 0.001; Study 5: *V* = 345.5, *p* < 0.001), and chose fewer LL options in the time-first condition (Study 2: mean = 0.527, SD = 0.270, *V* = 206.5, *p* < 0.001; Study 3: mean = 0.481, SD = 0.245, *V* = 159.5, *p* < 0.001; Study 4: mean = 0.548, SD = 0.290, *V* = 365, *p* < 0.001; Study 5: mean = 0.548, SD = 0.223, *V* = 393, *p* < 0.001; Fig. 3a–e; Supplementary Figs. 11–14).

However, there are potentially many reasons why our manipulation might have changed participants’ behavior. To verify that our manipulation affected attribute latency, and thus choice, we turn to Studies 4 and 5 and compare the MTTOL across conditions. Figure 3c, d show that, compared to the control condition, the attribute latency (MTTOL) increased in the amount-first condition and decreased in the time-first condition (two-sided Wilcoxon signed rank tests, Study 4: amount-first vs. control, *V* = 143, *p* < 0.001; control vs. time-first, *V* = 1492.5, *p* < 0.001; Study 5: amount-first vs. control, *V* = 257.5, *p* < 0.001; control vs. time-first, *V* = 1425, *p* = 0.004; see Supplementary Figs. 15 and 16 for details, and see Supplementary Figs. 17 and 18 for the correlations between MTTOL and the percentage of LL decisions within each condition). That is, compared to the control condition, displaying the amount information first induced participants to process the amount attribute earlier than the time attribute, while displaying the time information first induced participants to process the time attribute earlier than the amount attribute. The exogenous manipulation indeed altered participants’ attribute latencies in the expected way.

Moreover, Fig. 3c, d show that the behavioral change was in line with the MTTOL change across manipulation conditions (see Supplementary Figs. 13–16 for more details). The MTTOL change was correlated with the behavioral change across manipulation conditions in Study 5 (two-sided Spearman correlation tests, Fig. 3g: amount-first vs. time-first, *ρ* = 0.525, *p* < 0.001, *S* = 22772; Supplementary Fig. 20a: amount-first vs. control, *ρ* = 0.494, *p* < 0.001, *S* = 24264; Supplementary Fig. 20b: control vs. time-first, *ρ* = 0.330, *p* = 0.007, *S* = 32080). In Study 4, the MTTOL change was correlated with the behavioral change across amount-first and control conditions (Supplementary Fig. 19a, *ρ* = 0.382, *p* = 0.001, *S* = 33851) and across control and time-first conditions (Supplementary Fig. 19b, *ρ* = 0.406, *p* < 0.001, *S* = 27169), but not across amount-first and time-first conditions (Fig. 3f, *ρ* = 0.174, *p* = 0.165, *S* = 37792).

A mediation analysis based on bootstrapping (with 10,000 iterations) revealed that the 95% confidence interval for the indirect effect of manipulation on choice behavior through MTTOL did not include zero (Study 4: (0.058, 0.140), Study 5: (0.014, 0.090)). This indicates that the effect of manipulation on choice behavior was mediated via MTTOL (see Supplementary Figs. 21 and 22 for details).

The likely reason for the non-significant correlation between MTTOL change and behavioral change across amount-first and time-first conditions in Study 4 is that participants could choose before seeing the second attribute (i.e., before 3 s was up); this occurred in 45.3% of trials in the amount-first condition and in 17.2% of trials in the time-first condition. The IQR method eliminated most of the trials that were shorter than 3 s in the time-first condition, but not in the amount-first condition (see Methods for details). Supplementary Note 6 shows that the correlations between MTTOL change and behavioral change across time-first and amount-first conditions are significant if we exclude trials shorter than 3 s (Supplementary Fig. 23c), or if we keep trials shorter than 3 s and only exclude trials with extremely long RTs (Supplementary Fig. 24c). Thus, we are reasonably confident that the relationships between MTTOL change and behavioral change that we found in Study 5 (which sidesteps these issues), are also present in Study 4.

In summary, these results show that manipulating the order of displaying information alters participants’ attribute latencies and affects their choices. This confirms the causal role of attribute latency in shaping intertemporal decisions.

## Discussion

This paper describes the results of five separate studies designed to investigate the role of attribute latencies in determining intertemporal choices. We estimated attribute latencies using mouse-trajectory data and found that most (but not all) people process amount information before time information. These attribute latencies predicted individual differences in intertemporal choices, RTs, and behavioral changes across time constraints. We validated these results using an independent computational modeling analysis where the attribute latency was identified based on choice and RT data. Moreover, the behavioral changes under time constraints were in line with the attribute latency changes across participants. Then we tested whether exogenously manipulating the order of displaying amount and time information would influence attribute latency and causally change intertemporal preferences. Consistent with this hypothesis, our results revealed that manipulating the display order of the amount and time information altered the attribute latency and changed preferences. In particular, displaying amount before time makes people more patient, while displaying time before amount makes people less patient.

These results deepen our understanding of the nature of intertemporal decisions. The focus on intertemporal decisions is typically on just the choice itself, rather than the process underlying decisions. Researchers often treat observed differences in intertemporal choices as fixed differences in underlying parameters (e.g., discount rate) of the utility-based choice models. In contrast, we have shown that intra-choice dynamics, i.e., attribute latency, can explain individual differences in intertemporal choices and RTs. Because attribute latencies can depend on how choices are presented, this indicates that intertemporal preferences are not purely outcome-based.

We have shown that time pressure and delay have opposite effects on participants’ intertemporal preferences. The behavioral changes across time constraints cannot be parsimoniously explained by static utility models because those models do not account for the dynamics of the choice process. On the other hand, attribute latencies yield accurate predictions about the effects of time constraints on the direction and magnitude of behavioral changes.

Although dual-process theories are usually introduced to explain behavioral changes under time constraints, we offer an alternative explanation for such changes. Our results indicate that the attribute latency within a single evidence accumulation process might be a better explanation for the effects of time pressure on behavior. Prior work has also identified predispositions (i.e. starting-point biases) as an important factor in predicting the effects of time pressure^23^. Predispositions and attribute latencies both capture similar things, namely differences between the fastest and slowest decisions. Here (and elsewhere) there is evidence for both factors impacting choices^3,24,25,32^. In our study the stDDM did not significantly improve on the standard DDM in out-of-sample predictions. This is likely due to the high degree of mimicry between attribute latencies and predispositions. However, these two constructs are theoretically different and possible to distinguish in behavior. Predispositions occur prior to processing any information from the current choice problem and thus do not depend on trial-level variables. Attribute latencies capture a tendency to consider one attribute sooner than the other, and so their effects depend on trial-level attribute values^45^. It is still a debated issue how much the effects of time constraints on choices are due to predispositions^23^, attentional priorities^25,56^, or attribute latencies^45^. Future work will need to carefully disentangle these components of the decision process.

Different from Fisher^26^ which finds that manipulating exposure to the amount versus time information alters people’s patience while the order of displaying the two types of information has no significant effects on people’s choice behavior, the current study shows that simply manipulating the order of displaying information alters participants’ attribute latencies and affects their choices. The reason that there was no order effect in Fisher^26^ could be that this effect was dominated by the strong exposure manipulation.

The current study is also related to the research on order effects during information searching and choice tasks in marketing^57–59^, which are referred to as primacy and recency effects^60^. Studies in psychology and economics have explained these effects as decision-makers choosing the item they pay more attention to, and therefore choices can be manipulated via information search^61–64^. Here we reveal an underlying mechanism, attribute latency, that determines information processing order and can also be manipulated to influence choice behavior.

Many previous studies have neglected the role of individual differences and missed opportunities to link variability in the process to variability in behavior (Stillman et al.^36^) The current study seeks to address this issue by incorporating evidence related to heterogeneity across participants, linking aspects of the process-tracing data and model parameters to response latencies and choices. Future studies can examine how the manipulation interacts with people’s prior inclination to process attributes with different latencies. It would also be interesting to link the attribute latency with the actual timing with which the information is acquired using other process-tracing data, e.g., eye-tracking.

An important goal of this literature is the design of policies or interventions to ameliorate negative real-world outcomes. Knowing whether changes in attribute latencies causally influence intertemporal choices is useful because nudging might be easier than changing more hard-wired preference parameters^65^. Though intertemporal choice is not solely determined by the attribute latency, the current paper suggests that simply manipulating the attribute latency has substantial impacts on intertemporal choices. In particular, we show that manipulating the display order of the time and amount attributes is an effective way to manipulate participants’ intertemporal choices^66^. Moreover, policies of waiting periods (time delay) have been applied in real life to prompt a shift towards more deliberative thinking and lead to less myopic decisions^16,67,68^. Our results indicate that we should be careful when using such interventions since they might only be effective for some people, and push other people in the opposite direction.

## Methods

### Participants

The Institutional Review Board of the Neuromanagement Lab at Zhejiang University approved the experiment (Studies 1–5). 126 university students (70 females, mean age = 22.7 years, SD = 2.4 years) participated in Study 1 from June 2019 to June 2020 (interrupted by COVID-19), 49 students (29 females, mean age = 22.9 years, SD = 2.7 years) participated in Study 2 in April 2021, 43 students (22 females, mean age = 23.3 years, SD = 2.6 years) participated in Study 3 in May 2021, 69 students (43 females, mean age = 22.56 years, SD = 2.6 years) participated in Study 4 in January 2022, and 66 students (46 females, mean age = 21.62 years, SD = 2.1 years) participated in Study 5 in March 2022 at the Neuromanagement Lab, Zhejiang University. Informed consent was obtained from all participants before the experiment.

### Experimental design and procedure

Studies 1–5 used the same 300 trials. In these 300 trials, the payment times in the SS and LL options included today, 7 days, 30 days, 60 days, and 180 days. The reward amount in SS and LL options varied from 10 to 99 units. We provided participants with instructions before each block/condition. They could only start the experiment when they correctly answered the comprehension questions after reading the instructions. In Study 1, we tracked the mouse-trajectories of each decision using MouseTracker^37^ with a temporal resolution of 70 Hz. Participants were instructed to start moving the mouse as soon as the two options were displayed, and if they did not start to move the mouse in 750 ms, they were given a reminder message following that trial. In Studies 2 and 3 we programmed the experiment using Python and did not record the mouse-trajectories. The experiments in Studies 4 and 5 were also programed using Python and participants’ mouse-trajectories were recorded with a temporal resolution of 70 Hz. At the end of the experiment in each study, one trial was randomly selected for each participant and we transferred money to the participant via Alipay based on his/her decision on the date in the selected trial. On average, participants earned 56.5 RMB in Study 1, 55.4 RMB in Study 2, 61.0 RMB in Study 3, 63.3 RMB in Study 4, and 60.0 RMB in Study 5 (including the show-up fee of 15 RMB).

### Estimation of the mouse-trajectory-derived time-onset lag (MTTOL)

We normalized the coordinates of the center of the start box to $(0,0)$, and the center of the left and right options to $(-1,1)$ and $(1,1)$, respectively. Prior studies have often normalized the mouse trajectories into 100 intervals before performing analysis^38,44^. This might distort onset times because a unit of MTTOL in trials with longer durations is longer in absolute time than a unit of MTTOL in trials with shorter durations. Therefore, we extended the mouse-position at the last time point of each trial out to the maximum RT in each time condition^45^. Before doing this, we excluded trials with extremely long or short RTs using the IQR method (see Supplementary Fig. 3 for the correlations between the MTTOL based on the extended mouse-trajectory data and the MTTOL based on the raw mouse-trajectory data). That is, we eliminated trials where RTs were above the 0.75 quartile by more than 1.5 times the interquartile range, or below the 0.25 quantile by more than 1.5 times the interquartile range in each time condition. In Study 1, 6.2%, 0.02% and 7.8% of the trials were excluded from the time-free, time-pressure, and time-delay conditions, respectively. In Study 4 (5), 6.9% (6.5%), 1.0% (7.0%) and 22.2% (6.7%) of the trials were excluded from the control, amount-first, and time-first conditions, respectively. The reason that 22.2% of the trials were excluded from the time-first condition in Study 4 was that there were many trials faster than 3 s that were eliminated (see Supplementary Note 6). After this adjustment, all the mouse-trajectories in each condition had the same duration, i.e., the maximum RT of that condition. Then we equally split each extended trajectory into 100 intervals starting at time $t=1$ and ending at $t=101$. For each time $t$, we calculated the angle between the current cursor position and the start position (0, 0). Thus, an angle of $45^\circ /-45^\circ$ indicates direct movement toward the right/left option, and an angle of $0^\circ$ indicates direct movement upwards.

To identify the onset time that the participant started to consider and process each attribute, we estimated linear regressions at the participant level for how the trajectory angle at each time point $t$ was affected by the time (DiffTime = Time_right_ – Time_left_) and the amount differences (DiffAmount = Amount_right_ – Amount_left_) between the two options. The regression for participant ${i}$ at time point $t$ in each condition is:

1 $${{{{{{\rm{Angle}}}}}}}_{{itj}}={\gamma }_{{itc}}+{\gamma }_{{itT}}\times {{{{{{\rm{DiffTime}}}}}}}_{j}+{\gamma }_{{itA}}\times {{{{{{\rm{D}}}}}}{{{{{\rm{iff}}}}}}{{{{{\rm{Amount}}}}}}}_{j}$$

where ${\gamma }_{{itc}}$ is the constant, ${\gamma }_{{itT}}$ is the coefficient for the time difference, ${\gamma }_{{itA}}$ is the coefficient for the amount difference, and $j$ is the index of trials (observations). Consistent with Sullivan et al.^38^ and Lim et al.^44^, we carried out a one-tailed test of the hypothesis that the estimated regression coefficient of interest was significantly positive at the 5% level at time $t$. Using this procedure, we identified the earliest time $t$ at which the test was satisfied and remained significant until the end of the trial. We defined the difference between the onset times of the amount and time attributes as the attribute latency, i.e., mouse-trajectory-derived time-onset lag (MTTOL).

### Estimation of the starting-time drift diffusion model (stDDM)

We estimated the stDDM using the toolbox in Maier et al.^3^. The stDDM (Supplementary Fig. 4) allows one attribute to start affecting the drift rate later than the other. Specifically, if the amount attribute enters into the process first, the update equation of the relative evidence is:

2 $${R}_{t+1}={R}_{t}+\left({{\omega }_{c}+\left(t\, > \left|\frac{{{{{{\rm{TOL}}}}}}}{{{{{{\rm{d}}}}}}t}\right|\right)}\, * {\omega }_{T} * {{{{{\rm{DiffTime}}}}}}+{\omega }_{A} * {{{{{\rm{DiffAmount}}}}}}\right) * {{{{{\rm{d}}}}}}t+\varepsilon$$

If the time attribute enters into the process first, the update equation of the relative evidence is:

3 $${R}_{t+1}={R}_{t}+\left({{\omega }_{c}+\omega }_{T} * {{{{{\rm{DiffTime}}}}}}+{\left(t\, > \left|\frac{{{{{{\rm{TOL}}}}}}}{{{{{{\rm{d}}}}}}t}\right|\right)} * { \omega }_{A} * {{{{{\rm{DiffAmount}}}}}}\right) * {{{{{\rm{d}}}}}}t+\varepsilon$$

where TOL (time-onset lag) is the onset time that the amount attribute begins to affect the decision process minus the onset time that the time attribute begins to affect it, and $\varepsilon$ represents the amount of zero-mean Gaussian noise. In addition to these drift-rate parameters, the stDDM includes three additional parameters for: (1) Boundary separation ($a$), (2) non-decision time (${t}_{0}$), and (3) starting point ($z$). Without loss of generality, we fixed the noise parameter ($\varepsilon$) to 1 in the estimation. In the estimation, we coded the decision of choosing the SS option as 1 and the decision of choosing the LL option as 0. That is, a starting point greater than 0.5 represents a predisposition towards the SS option, and a starting point less than 0.5 represents a predisposition towards the LL option.

### Reporting summary

Further information on research design is available in the Nature Portfolio Reporting Summary linked to this article.

### Supplementary information

Supplementary Information

Reporting Summary

## Data Availability

The data generated in this study have been deposited to the Open Science Framework (10.17605/OSF.IO/CY4GR).

## Code availability

The code for the analyses presented in this article have been deposited to the Open Science Framework (10.17605/OSF.IO/CY4GR).
