# Supplementary material for: Attribute latencies causally shape intertemporal decisions
Source: Nat Commun. 2024 Apr 5;15:2948. doi: 10.1038/s41467-024-46657-2 (PMC10997753; doi:10.1038/s41467-024-46657-2)
Supplement: Supplementary file 1 — Supplementary Information [file 41467_2024_46657_MOESM1_ESM.pdf]

Supplementary Information for

"Attribute Latencies Causally Shape Intemporal Decisions"

Fadong Chen, Jiehui Zheng, Lei Wang, Ian Krajbich

Supplementary Note 1

Mouse-trajectory-derived time-onset lag (MTTOL) in Study 1

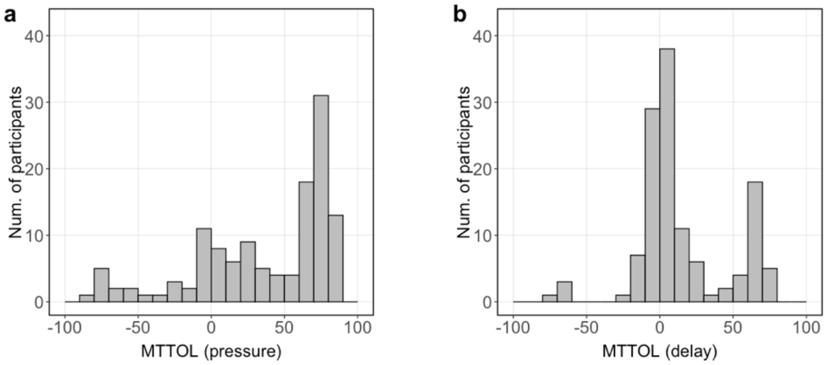

Supplementary Fig. 1. Distributions of the mouse-trajectory-derived time-onset lag (MTTOL) in the time-pressure (a) and delay (b) conditions of Study 1.

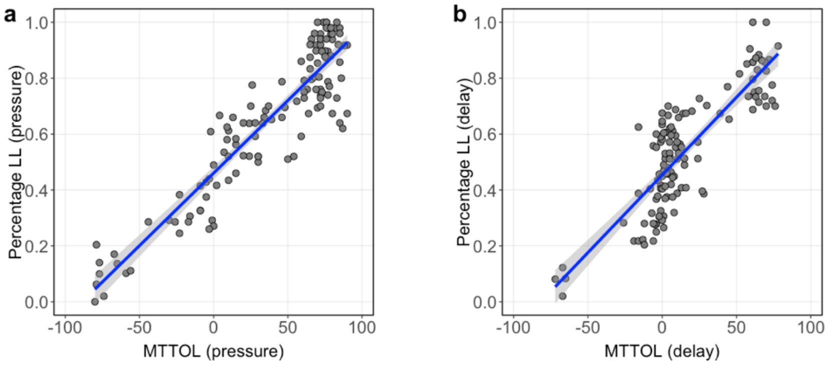

Supplementary Fig. 2. Correlations between the percentage of LL decisions and the mouse-trajectory-derived time-onset lag (MTTOL) in the time-pressure (a) and delay (b) conditions of Study 1. Each dot represents one participant, the blue solid lines and the gray shadings are the fitted linear regression lines and their standard errors.

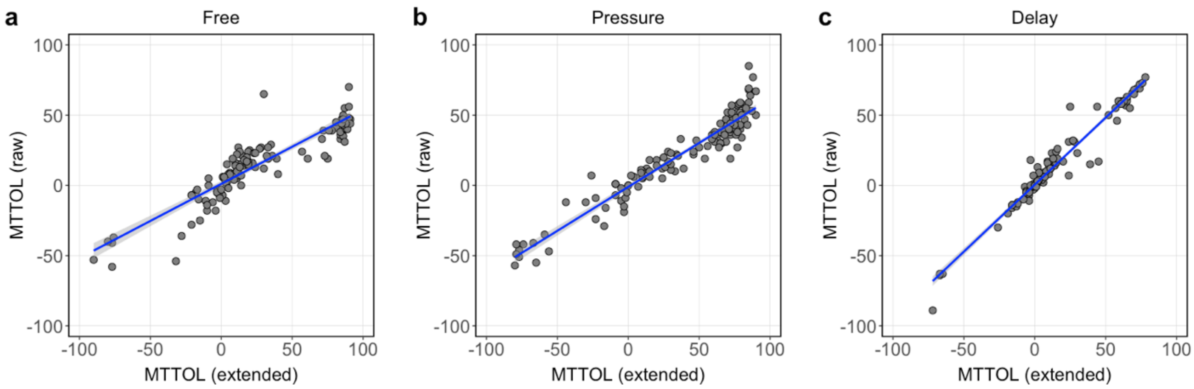

Supplementary Fig. 3. Correlations between the MTTOL based on the extended mouse-trajectory data and the MTTOL based on the raw data in the time-free (a), pressure (b), and delay (c) conditions. The MTTOL on the *x*-axis was estimated using the extended mouse-trajectory data where we extended the last point of each trial to the maximum response time in each condition. The MTTOL on the *y*-axis was estimated using the raw mouse-trajectory data. Each dot represents one participant, the blue solid lines and the gray shadings are the linear regression lines and their standard errors. Two-sided Pearson correlation tests, free: $r(124) = 0.907$, $p < 0.001$, $t = 24.027$, 95% CI = [0.871, 0.934]; pressure: $r(124) = 0.958$, $p < 0.001$, $t = 37.043$, 95% CI = [0.940, 0.970]; delay: $r(124) = 0.979$, $p < 0.001$, $t = 53.087$, 95% CI = [0.970, 0.985].

Supplementary Note 2

Computational modeling analysis for Study 1

2.1 Estimation of the starting-time drift-diffusion model (stDDM)

Supplementary Fig. 4 shows a graphical illustration of the starting-time drift diffusion model (stDDM). We estimated the stDDM hierarchically for the time-free condition both at the aggregate and participant level. Supplementary Table 1 displays the estimation results at the aggregate level.

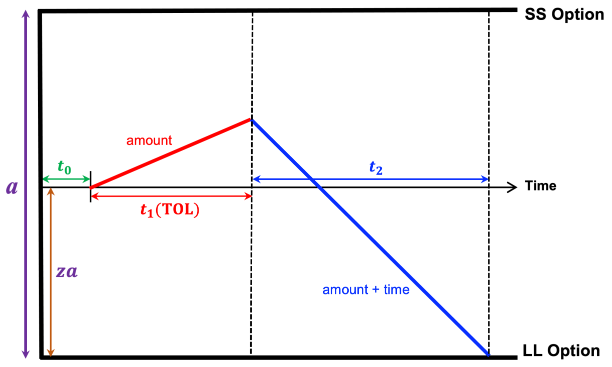

Supplementary Fig. 4. A graphical illustration of the starting-time drift diffusion model (stDDM). a denotes the boundary separation, t_0 the non-decision time, and z the starting point (which indicates the prior bias towards the SS option ($z > 0.5$) or the LL option ($z < 0.5$)). The red and blue trajectory displays an example of the evolution of the relative evidence. In the example, the amount attribute enters into the evidence accumulation process first at t_0 and the time attribute enters into the process later at time t_0+t_1. We refer to the duration of t_1 as the time-onset lag (TOL), which in this case is positive. For illustrative purposes, here we have omitted the diffusion noise in the process and only shown the average drift rates.

Supplementary Table 1. The estimation result of the stDDM at the aggregate level

Parameter	Mean (95% HDI)
z (starting point)	0.483 (0.469, 0.499)
$RTTOL$ (response-time-derived time-onset lag)	0.330 (0.264, 0.399)
ω_0 (drift-rate constant)	0.098 (-0.071, 0.272)
ω_T (subjective weight on the time attribute)	0.010 (0.003, 0.017)
ω_A (subjective weight on the amount attribute)	-0.038 (-0.045, -0.030)
t_0 (non-decision time)	0.844 (0.805, 0.884)
a (threshold)	2.936 (2.829, 3.049)

2.2 Parameter recovery exercise for the starting-time drift diffusion model (stDDM)

In this parameter recovery exercise, we generated simulated choices and response times (RTs) by parameterizing the stDDM using the best fitting parameters in Study 1. The simulated choice sets were based on these parameters and the differences in the time and amount attributes that participants faced in each trial of the time-free condition in Study 1. Thus, the simulated choice sets matched the experimental data in terms of trial numbers and attribute differences. Fitting these simulated choices allowed us to quantify the stDDM's ability to recover known parameter values within the context of our experimental settings and the ability to distinguish components in the sequential sampling process underlying decisions.

The parameter recovery exercise yielded accurate parameter estimates. The recovered parameters were highly correlated with the generating parameters, with the only exception being the starting-point parameters, which were only moderately correlated with each other (Supplementary Fig. 5, two-sided Pearson correlation tests, $r(124) = 0.979$, $p < 0.001$, $t = 53.764$, 95% CI = [0.971, 0.985] for the drift-rate constant; $r(124) = 0.958$, $p < 0.001$, $t = 37.098$, 95% CI = [0.940, 0.970] for the subjective weight on the time attribute; $r(124) = 0.913$, $p < 0.001$, $t = 24.902$, 95% CI = [0.878, 0.938] for the subjective weight on the amount attribute; $r(124) = 0.929$, $p < 0.001$, $t = 27.936$, 95% CI = [0.900, 0.950] for the attribute latency (time-onset lag, TOL); $r(124) = 0.419$, $p < 0.001$, $t = 5.133$, 95% CI = [0.263, 0.553] for the starting point (predisposition); $r(124) = 0.895$, $p < 0.001$, t = 22.388, 95% CI = [0.854, 0.925] for the threshold; $r(124) = 0.975$, $p < 0.001$, $t = 48.983$, 95% CI = [0.965, 0.982] for the non-decision time).

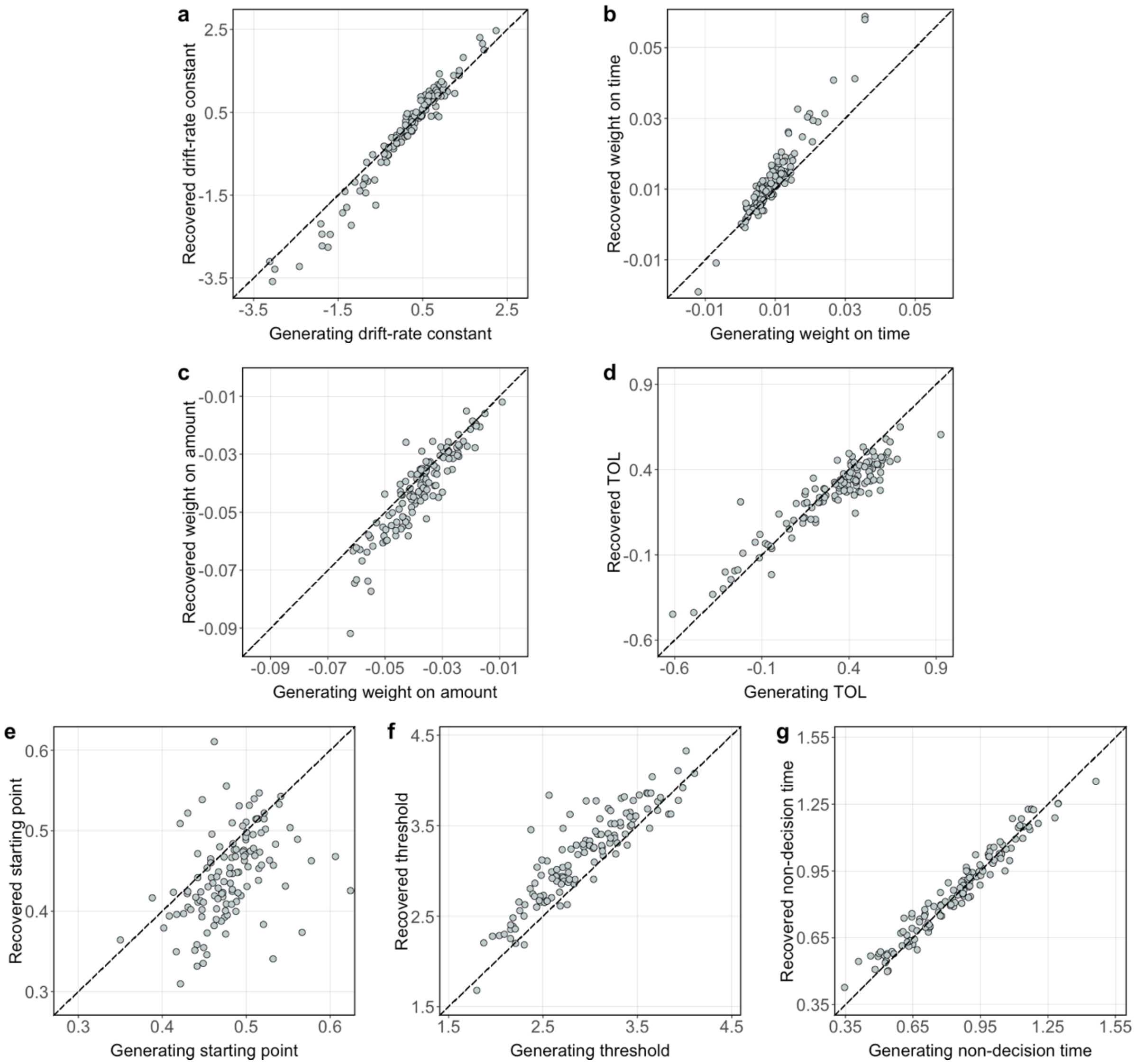

Supplementary Fig. 5. Parameter recovery exercise for the stDDM: generating parameters vs. recovered parameters. (a) Drift-rate constant; (b) Subjective weight on the time attribute; (c) Subjective weight on the amount attribute; (d) Time-onset lag (TOL, attribute latency); (e) Starting point; (f) Threshold; (g) Non-decision time. Each dot represents one participant. The dashed lines are the identity lines.

2.3 Model comparison

In addition to the stDDM (Model 1, M1, see Supplementary Fig. 4), we fit four other versions of the DDM to the time-free data in Study 1. Model 2 (standard DDM, M2) is the standard DDM without attribute latency. That is, both the amount and the time attributes enter into the evidence accumulation process simultaneously. Model 3 (simple stDDM, M3) is a version of the stDDM without the starting-point bias and the drift-rate constant. That is, the starting point is 0.5 and the drift-rate constant is 0 in the simple stDDM. Model 4 (vspDDM, M4) is a version of the standard DDM with a varying starting point, i.e., the starting point is a linear function of the attribute differences. Model 5 (simple DDM, M5) is a version of the standard DDM without the starting-point bias and the drift-rate constant (analogous to Model 3). We estimated the vspDDM (M4) using the HDDM toolbox [1], and the other three models using the same toolbox as the stDDM.

Since the five models were estimated using different methods and these models have different number of parameters, we compared the out-of-sample predictive power of these models. Specifically, we estimated each model at the participant level using half of the time-free trials, and used the estimates to predict participants' choices in the other half of the time-free trials. We used Cramer's λ [2,3,4] to measure the predictive performance of each model.

Supplementary Table 2 shows the summary of the Cramer's λ for these five models. The Cramer's λ of stDDM was higher but non-significant than that of the standard DDM (two-sided Wilcoxon signed rank tests, $V = 3380$, $p = 0.770$). Both the Cramer's λ of stDDM and the standard DDM were greater than those of the other models (stDDM vs. simple stDDM, $V = 5051$, $p = 0.011$; stDDM vs. vspDDM, $V = 6755$, $p < 0.001$; stDDM vs. simple DDM, $V = 6356$, $p < 0.001$; standard DDM vs. vspDDM, $V = 6436$, $p < 0.001$; standard DDM vs. simple DDM, $V = 6226$, $p < 0.001$) except standard DDM versus simple stDDM ($V = 4802$, $p = 0.051$). Therefore, we focused on the two best models, the stDDM and the standard DDM, in the main text.

Supplementary Table 2. Cramer's λ for the five versions of the drift diffusion models

Models		Cramer's λ
M1	stDDM	0.425 (0.017)
M2	standard DDM	0.418 (0.018)
M3	simple stDDM	0.405 (0.014)
M4	vspDDM	0.399 (0.016)
M5	simple DDM	0.378 (0.015)

Notes: Each model was estimated using one half of the time-free trials at the participant level, and the estimates were used to predict participants' choices in the other half of the time-free trials in Study 1. The standard errors of the mean were reported in parentheses.

2.4 Attribute latency (time-onset lag, TOL) predicts individual differences in choices and RTs

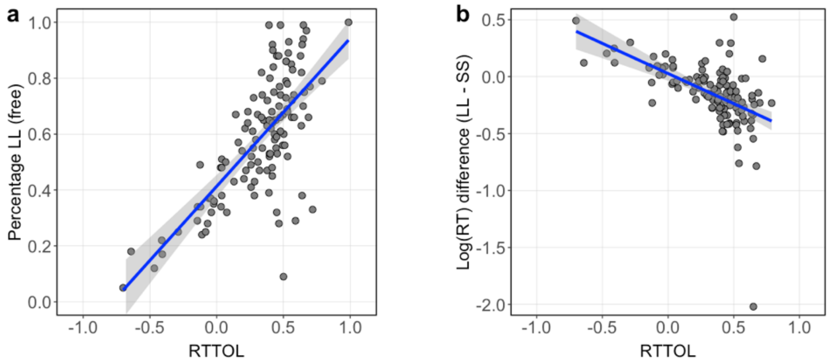

Supplementary Fig. 6. Correlations between the RTTOL (response-time-derived time-onset lag) in the stDDM (starting-time drift diffusion model) estimated using one half of the time-free trials and the percentage of LL decisions (a) (two-sided Pearson correlation test, $r(124) = 0.709$, $p < 0.001$, $t = 11.186$, 95% CI = [0.609, 0.786]) and the log(RT) differences between LL and SS decisions (b) ($r(123) = -0.535$, $p < 0.001$, $t = -7.018$, 95% CI = [-0.649, -0.396]) computed using the other half of the time-free trials. Each dot represents one participant, the blue solid lines and the gray shadings are the fitted linear regression lines and their standard errors.

Supplementary Note 3

Attribute latency adds additional power in explaining choices and response times

Supplementary Table 3. OLS regressions of the percentage of LL decisions on the MTTOL, stDDM and standard DDM parameters in the time-free condition of Study 1

	(1)				(2)				(3)				(4)			
	stDDM				stDDM				stDDM				Standard DDM			
	Coef.	SE	*t*	*p*	Coef.	SE	*t*	*p*	Coef.	SE	*t*	*p*	Coef.	SE	*t*	*p*
Constant	0.747	0.064	11.670	<0.001	0.959	0.096	9.999	<0.001	0.723	0.079	9.118	<0.001	0.949	0.056	16.884	<0.001
z	-0.529	0.126	-4.195	<0.001	-0.683	0.194	-3.514	<0.001	-0.501	0.153	-3.278	0.001	-0.945	0.116	-8.124	<0.001
ω_c	-0.242	0.013	-17.946	<0.001	-0.340	0.017	-19.709	<0.001	-0.229	0.018	-12.434	<0.001	-0.284	0.008	-34.444	<0.001
RTTOL	0.268	0.021	12.948	<0.001												
MTTOL									0.002	0.0002	8.842	<0.001				
ω_T	-6.015	1.440	-4.178	<0.001	-14.676	1.975	-7.341	<0.001	-10.709	1.602	-6.684	<0.001	-20.646	1.428	-14.457	<0.001
ω_A	-5.320	0.439	-12.131	<0.001	-5.760	0.677	-8.503	<0.001	-4.458	0.548	-8.140	<0.001	-6.326	0.404	-15.674	<0.001
t_0	0.022	0.022	0.981	0.328	0.005	0.034	0.132	0.896	0.036	0.027	1.325	0.188	0.057	0.026	2.172	0.032
a	-0.003	0.010	-0.357	0.722	-0.015	0.015	-1.016	0.311	0.007	0.012	0.636	0.526	0.010	0.010	1.022	0.309
Adj. R^2	0.945				0.868				0.920				0.938			
Num. obs.	126				126				126				126			

Notes: The dependent variable is the percentage of LL decisions in the time-free condition. The percentage of LL decisions is computed using half of the time-free trials in Study 1, the MTTOL, the stDDM and the standard DDM are estimated using the other half of the time-free trials.
Abbreviations: z is the starting point, ω_c is the drift-rate constant, ω_T is the subjective weight on the time attribute, ω_A is the subjective weight on the amount attribute, t_0 is the non-decision time, a is the threshold, *RTTOL* is the response-time-derived time-onset lag, and *MTTOL* is the mouse-trajectory-derived time-onset lag.
SE indicates the standard error, *t* indicates t-value, *p* indicates p-value, and p-values are two-sided.

Supplementary Table 4. OLS regressions of the RT differences between LL and SS decisions on the MTTOL, the stDDM and standard DDM parameters in the time-free condition of Study 1

	(1)				(2)				(3)				(4)			
	stDDM				stDDM				stDDM				Standard DDM			
	Coef.	SE	*t*	*p*	Coef.	SE	*t*	*p*	Coef.	SE	*t*	*p*	Coef.	SE	*t*	*p*
Constant	-1.099	0.233	-4.724	<0.001	-1.405	0.246	-5.700	<0.001	-1.107	0.249	-4.445	<0.001	-1.355	0.197	-6.875	<0.001
z	2.470	0.456	5.412	<0.001	2.694	0.498	5.404	<0.001	2.463	0.479	5.139	<0.001	2.744	0.406	6.753	<0.001
ω_c	0.143	0.049	2.939	0.004	0.279	0.044	6.301	<0.001	0.137	0.058	2.369	0.019	0.207	0.029	7.076	<0.001
$RTTOL$	-0.376	0.075	-5.014	<0.001												
$MTTOL$									-0.003	0.001	-3.593	<0.001				
ω_T	-3.810	5.206	-0.732	0.466	8.282	5.063	1.636	0.105	3.195	5.029	0.635	0.527	16.170	4.994	3.238	0.002
ω_A	4.282	1.596	2.683	0.008	4.977	1.745	2.852	0.005	3.344	1.724	1.940	0.055	5.144	1.430	3.597	<0.001
t_0	0.230	0.080	2.869	0.005	0.253	0.088	2.883	0.005	0.213	0.084	2.523	0.013	0.228	0.090	2.526	0.013
a	-0.106	0.035	-3.034	0.003	-0.088	0.038	-2.296	0.023	-0.116	0.037	-3.106	0.002	-0.089	0.034	-2.644	0.009
Adj. R^2	0.573				0.486				0.533				0.553			
Num. obs.	125				125				125				125			

Notes: The dependent variable is the RT differences between LL and SS choices in the time-free condition. The RT difference is computed using half of the time-free trials, the stDDM and the standard DDM are estimated using the other half of the time-free trials. There was one participant who always chose LL options. Thus, we have 125 participants left in regressions.
Abbreviations: z is the starting point, ω_c is the drift-rate constant, ω_T is the subjective weight on the time attribute, ω_A is the subjective weight on the amount attribute, t_0 is the non-decision time, a is the threshold, $RTTOL$ is the response-time-derived amount-onset lag, and $MTTOL$ is the mouse-trajectory-derived time-onset lag.
SE indicates the standard error, *t* indicates t-value, *p* indicates p-value, and p-values are two-sided.

Supplementary Note 4

Intertemporal choice behavior across time conditions in Study 1

4.1 Percentage of choosing LL options across time conditions

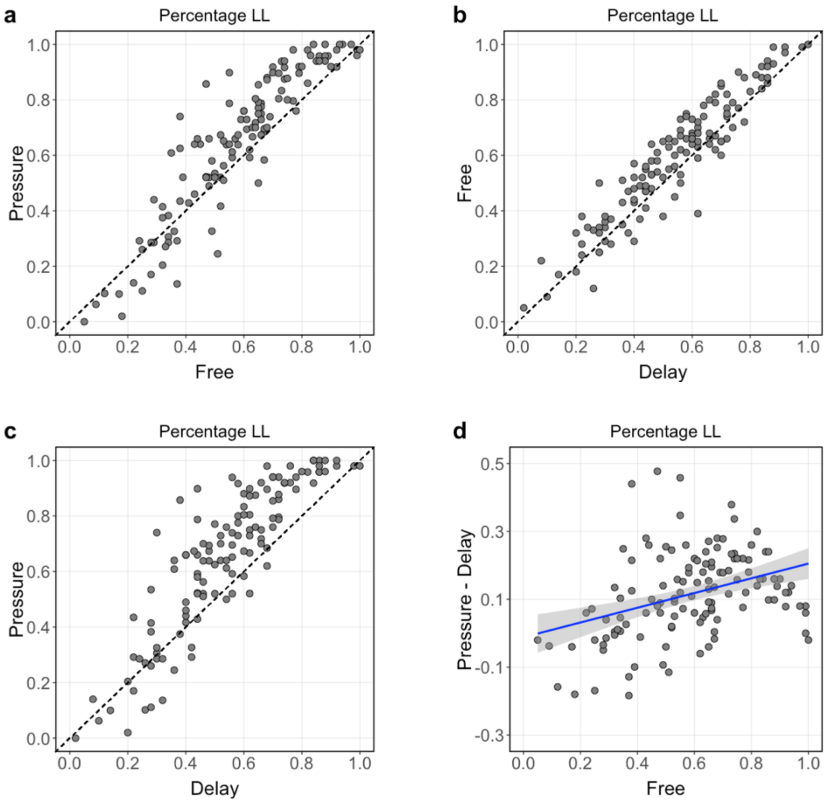

Supplementary Fig. 7. **Percentage of LL decisions across time conditions.** (a) Pressure vs. Free; (b) Free vs. Delay; (c) Pressure vs. Delay; (d) (Pressure—Delay) vs. Free. Each dot represents a participant. The dashed lines in (a)-(c) are the identity lines, the blue solid line in (d) is the fitted linear regression line and the gray shading is the standard error.

4.2 The attribute latency (mouse-trajectory-derived time-onset lag, MTTOL) in the time-free condition predicts behavioral changes across time conditions

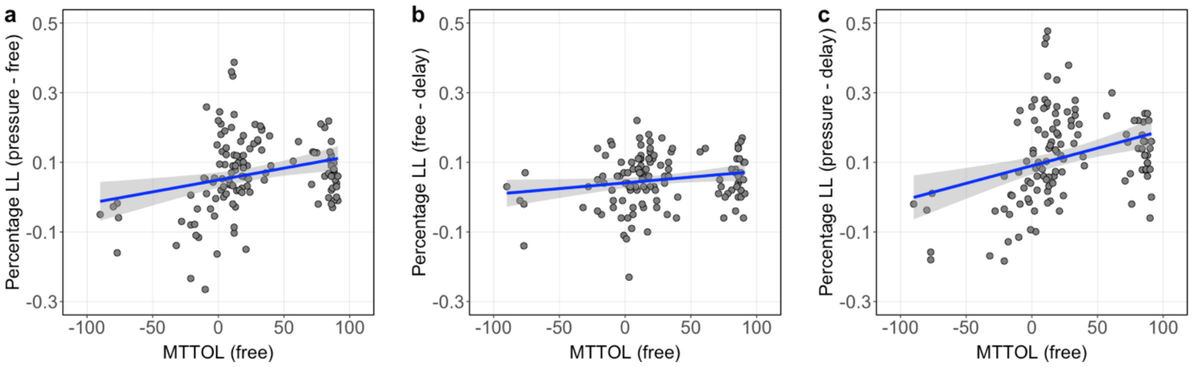

Supplementary Fig. 8. **Correlations between the MTTOL (mouse-trajectory-derived time-onset lag) in the time-free condition and the behavioral changes across time conditions.** (a) MTTOL in the time-free condition vs. Differences in the percentage of LL decisions across time-pressure and free conditions (two-sided Spearman correlation test, ρ = 0.280, p = 0.001, S = 239967). (b) MTTOL in the time-free condition vs. Differences in the percentage of LL decisions across time-free and delay conditions (ρ = 0.188, p = 0.035, S = 270766). (c) MTTOL in the time-free condition vs. Differences in the percentage of LL decisions across time-pressure and delay conditions (ρ = 0.377, p < 0.001, S = 207717)). The MTTOL was estimated using one half of the time-free trials in Study 1, and the percentage of LL decisions in the time-free condition was computed using the other half of the time-free trials. Each dot represents one participant, the blue solid lines and the gray shadings are the fitted linear regression lines and their standard errors.

The OLS regressions in Supplementary Table 5 show that, except RTTOL, all the other parameters in stDDM explained 12.8% of the variance of behavioral changes across time-pressure and delay conditions (model 2). Adding the MTTOL (model 3) significantly increases R^2 from 0.128 to 0.172 (models 2 vs. 3, two-sided partial-F test, F-value = 7.257, p = 0.008). The R^2 of the regression on standard DDM parameters (0.165) is less than that of stDDM parameters (0.187, models 1 vs. 4, two-sided partial-F test, F-value = 4.172, p = 0.043). Thus, taking attribute latency into account can better explain the directions and magnitudes of behavioral changes across time conditions.

Supplementary Table 5. OLS regressions of behavioral changes across time-pressure and delay conditions on the MTTOL, the stDDM and the standard DDM parameters

	(1)				(2)				(3)				(4)			
	stDDM				stDDM				stDDM				Standard DDM			
	Coef.	SE	*t*	*p*	Coef.	SE	*t*	*p*	Coef.	SE	*t*	*p*	Coef.	SE	*t*	*p*
Constant	0.166	0.145	1.143	0.255	0.282	0.146	1.933	0.056	0.145	0.151	0.962	0.338	0.193	0.122	1.580	0.117
z	-0.234	0.287	-0.817	0.415	-0.318	0.295	-1.708	0.283	-0.213	0.291	-0.732	0.466	-0.345	0.253	-1.364	0.175
ω_c	-0.071	0.031	-2.326	0.022	-0.125	0.026	-4.759	<0.001	-0.060	0.035	-1.724	0.087	-0.046	0.018	-2.541	0.012
$RTTOL$	0.146	0.047	3.102	0.002												
$MTTOL$									0.001	0.0005	2.694	0.008				
ω_T	-6.912	3.272	-2.112	0.037	11.619	3.000	-3.876	<0.001	-9.331	3.046	-3.063	0.003	-6.124	3.104	-1.973	0.051
ω_A	-3.338	0.007	-3.349	0.001	-3.578	1.029	-3.477	<0.001	-2.824	1.041	-2.712	0.008	-3.887	0.877	-4.431	<0.001
t_0	0.056	0.050	1.111	0.269	0.047	0.052	0.894	0.373	0.065	0.051	1.262	0.209	0.048	0.056	0.845	0.400
a	-0.011	0.022	-0.509	0.611	-0.017	0.022	-0.775	0.440	-0.004	0.022	-0.195	0.846	0.015	0.021	0.702	0.484
Adj. R^2	0.187				0.128				0.172				0.165			
Num. obs.	126				126				126				126			

Notes: The dependent variable is the percentage changes of choosing LL options across time-pressure versus delay conditions.
Abbreviations: z is the starting point, ω_c is the drift-rate constant, ω_T is the subjective weight on the time attribute, ω_A is the subjective weight on the amount attribute, t_0 is the non-decision time, a is the threshold, $RTTOL$ is the response-time-derived time-onset lag, and $MTTOL$ is the mouse-trajectory-derived time-onset lag.
SE indicates the standard error, *t* indicates t-value, *p* indicates p-value, and p-values are two-sided.

4.3 The attribute latency (mouse-trajectory-derived time-onset lag, MTTOL) across time-pressure and free conditions

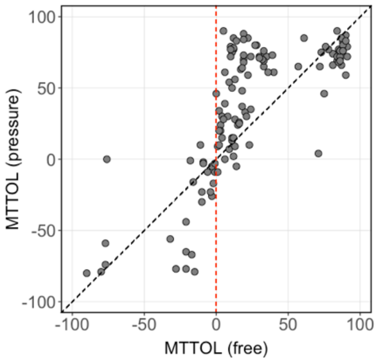

Supplementary Fig. 9. **The attribute latency (mouse-trajectory-derived time-onset lag, MTTOL) changes across time-free and pressure conditions.** Each dot represents one participant. The black dashed line is the identity line, and the vertical red dashed line splits participants into two groups, one with positive MTTOL and the other with negative MTTOL. For the 26 participants whose MTTOL was negative in the time-free condition, their MTTOL in the time-pressure condition was less (but not significant) than their MTTOL in the time-free condition (two-sided Wilcoxon signed rank test, $V = 231.5$, $p = 0.065$), while for the 99 participants whose MTTOL was positive in the time-free condition, their MTTOL in the time-pressure condition was greater than their MTTOL in the time-free condition ($V = 1148$, $p < 0.001$). That is, time pressure sped up the processing of one attribute over the other.

4.4 Mediation analysis for Study 1

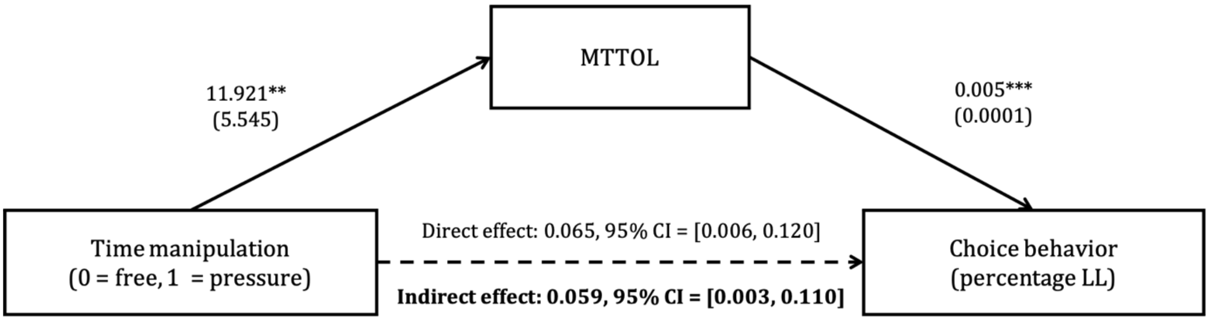

Supplementary Fig. 10. **Mediation analysis for Study 1.** The effect of time constraint on choice behavior was mediated via MTTOL. Model tests the indirect effect of time constraint (0 = free, 1 = pressure) on choice behavior (percentage of LL choices) through MTTOL by using a 95% confidence interval with 10,000 bootstrapped samples. The confidence interval for the indirect effect does not cross zero, indicating significant mediation. *p<0.1, **p<0.05, ***p<0.01.

Supplementary Note 5

Manipulating intertemporal choice behavior via attribute latency

5.1 Percentage of choosing LL options across manipulation conditions

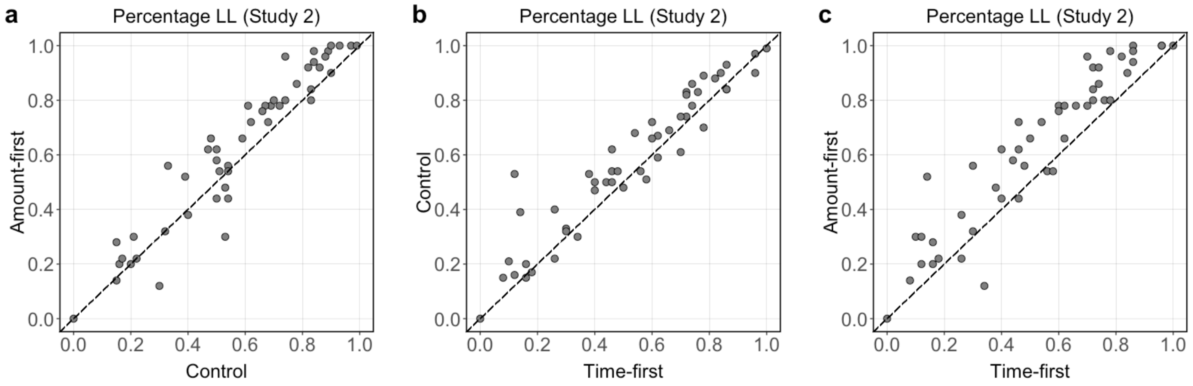

Supplementary Fig. 11. **Percentage of LL decisions across manipulation conditions in Study 2.** (a) Amount-first vs. Control (two-sided Wilcoxon signed rank test, $V = 151$, $p < 0.001$); (b) Control vs. Time-first ($V = 206.5$, $p < 0.001$); (c) Amount-first vs. Time-first ($V = 70.5$, $p < 0.001$). Each dot represents one participant, and the dashed lines are the identity lines.

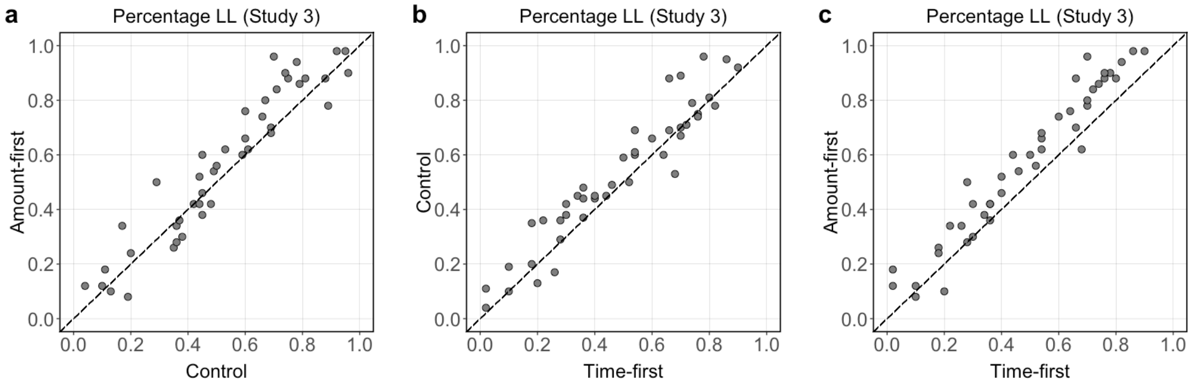

Supplementary Fig. 12. **Percentage of LL decisions across manipulation conditions in Study 3.** (a) Amount-first vs. Control (two-sided Wilcoxon signed rank test, $V = 222.5$, $p = 0.007$); (b) Control vs. Time-first ($V = 159.5$, $p < 0.001$); (c) Amount-first vs. Time-first ($V = 27.5$, $p < 0.001$). Each dot represents one participant, and the dashed lines are the identity lines.

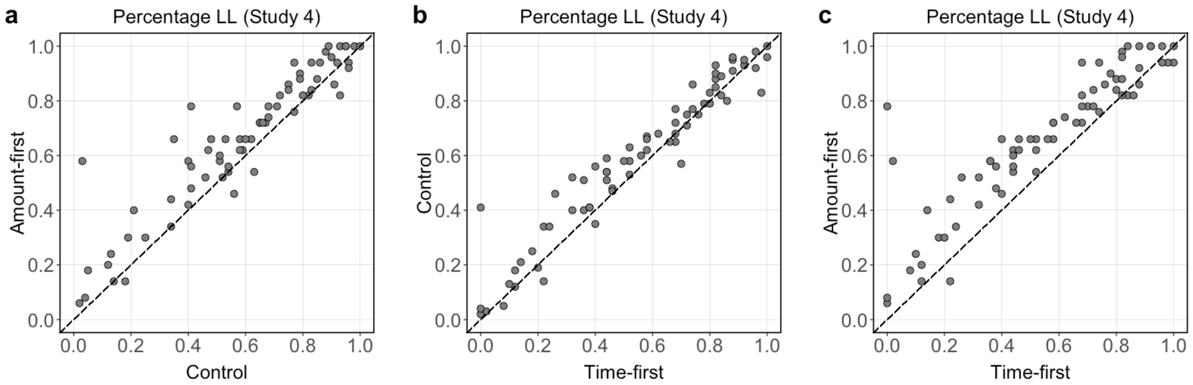

Supplementary Fig. 13. Percentage of LL decisions across manipulation conditions in Study 4. (a) Amount-first vs. Control (two-sided Wilcoxon signed rank test, $V = 180$, $p < 0.001$); (b) Control vs. Time-first ($V = 365$, $p < 0.001$); (c) Amount-first vs. Time-first ($V = 66$, $p < 0.001$). Each dot represents one participant, and the dotted dashed lines are the identity lines.

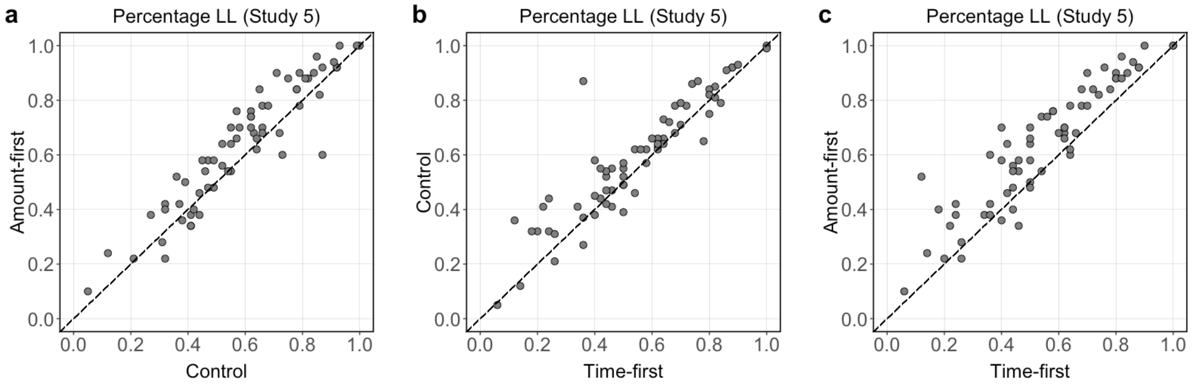

Supplementary Fig. 14. Percentage of LL decisions across manipulation conditions in Study 5. (a) Amount-first vs. Control (two-sided Wilcoxon signed rank test, $V = 345.5$, $p < 0.001$); (b) Control vs. Time-first ($V = 393$, $p < 0.001$); (c) Amount-first vs. Time-first ($V = 96.5$, $p < 0.001$). Each dot represents one participant, and the dashed lines are the identity lines.

5.2 The attribute latency (mouse-trajectory-derived time-onset lag, MTTOL) across manipulation conditions in Studies 4 and 5

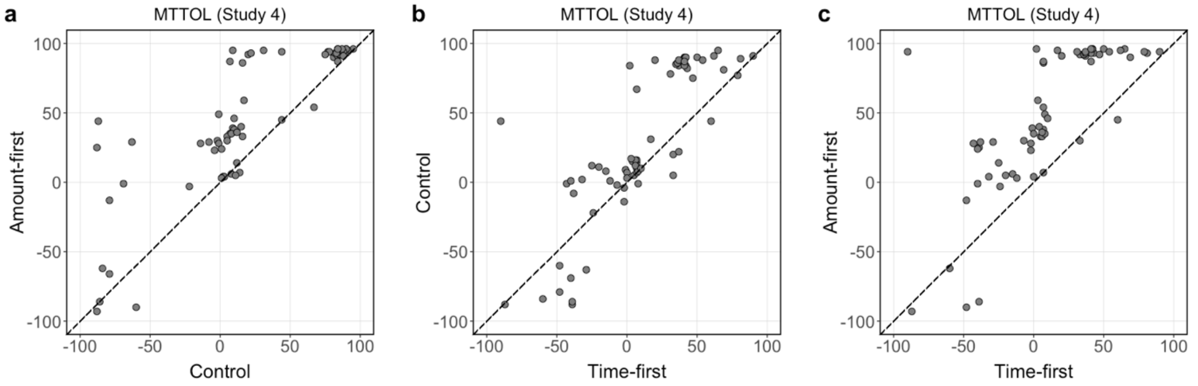

Supplementary Fig. 15. The attribute latency (MTTOL) across manipulation conditions in Study 4. (a) Amount-first vs. Control (two-sided Wilcoxon signed rank tests, $V = 143$, $p < 0.001$); (b) Control vs. Time-first ($V = 1492.5$, $p < 0.001$); (c) Amount-first vs. Time-first ($V = 1991.5$, $p < 0.001$). Each dot represents one participant, and the dashed lines are the identity lines.

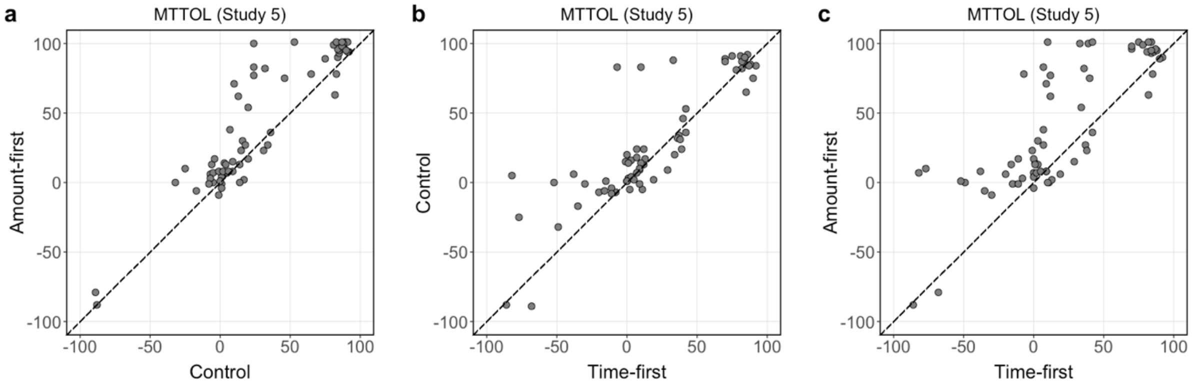

Supplementary Fig. 16. The attribute latency (MTTOL) across manipulation conditions in Study 5. (a) Amount-first vs. Control (two-sided Wilcoxon signed-rank tests, $V = 257.5$, $p < 0.001$); (b) Control vs. Time-first ($V = 1425$, $p = 0.004$); (c) Amount-first vs. Time-first ($V = 1934.5$, $p < 0.001$). Each dot represents one participant, and the dashed lines are the identity lines.

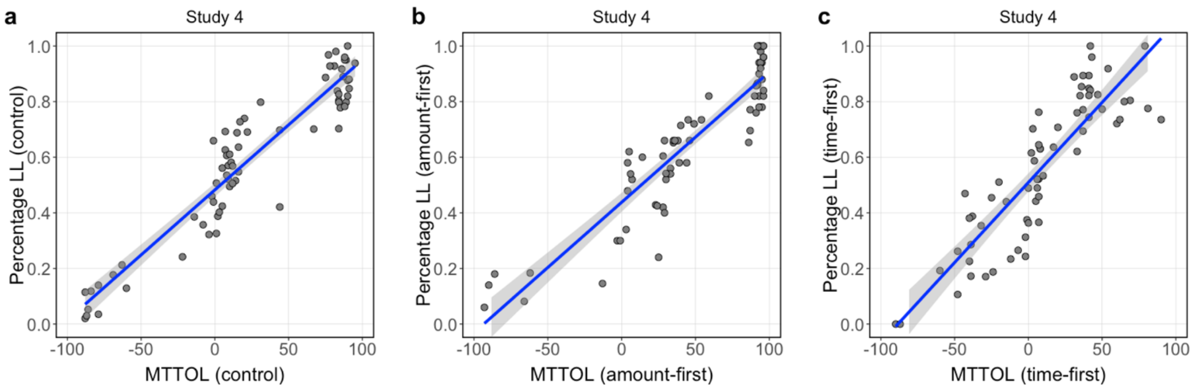

Supplementary Fig. 17. Correlations between the percentage of LL decisions and the attribute latency (mouse-trajectory-derived time-onset lag, MTTOL) within each condition in Study 4. (a) Percentage of LL decisions vs. MTTOL in the control condition (two-sided Pearson correlation test, $r(67) = 0.943$, $p < 0.001$, $t = 23.251$, 95% CI = [0.910, 0.965]); (b) Percentage of LL decisions vs. MTTOL in the amount-first condition ($r(67) = 0.919$, $p < 0.001$, $t = 19.139$, 95% CI = [0.873, 0.949]); (c) Percentage of LL decisions vs. MTTOL in the time-first condition ($r(63) = 0.873$, $p < 0.001$, $t = 14.208$, 95% CI = [0.799, 0.921]). The percentage of LL decisions in the control condition was computed using one half of the trials in the control condition in Study 4, and the MTTOL in the control condition was estimated using the mouse-tracking data in the other half of the trials in the control condition. Each dot represents one participant, the blue solid lines and the gray shadings are the fitted linear regression lines and their standard errors.

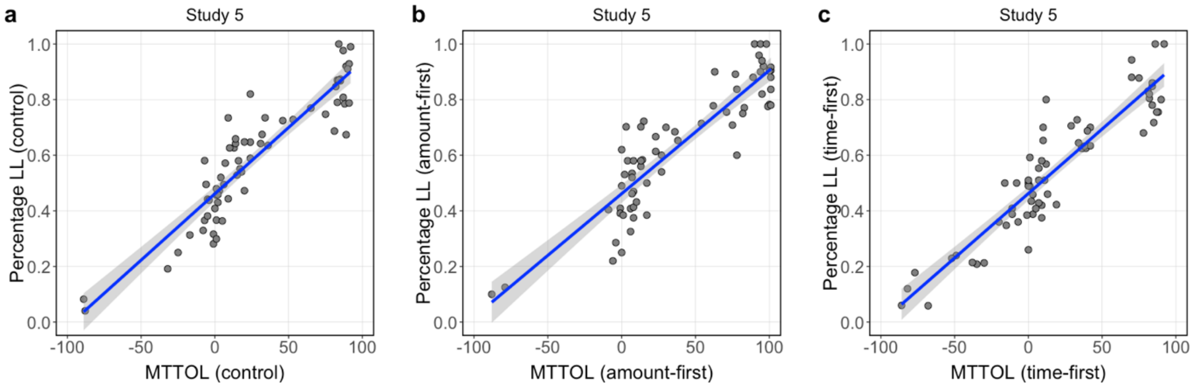

Supplementary Fig. 18. Correlations between the percentage of LL decisions and the attribute latency (mouse-trajectory-derived time-onset lag, MTTOL) within each condition in Study 5. (a) Percentage of LL decisions vs. MTTOL in the control condition (two-sided Pearson correlation test, $r(64) = 0.912$, $p < 0.001$, $t = 17.778$, 95% CI = [0.860, 0.945]); (b) Percentage of LL decisions vs. MTTOL in the amount-first condition ($r(64) = 0.899$, $p < 0.001$, $t = 16.396$, 95% CI = [0.839, 0.937]); (c) Percentage of LL decisions vs. MTTOL in the time-first condition ($r(64) = 0.921$, $p < 0.001$, $t = 18.912$, 95% CI = [0.874, 0.951]). The percentage of LL decisions in the control condition was computed using one half of the trials in the control condition in Study 5, and the MTTOL in the control condition was estimated using the mouse-tracking data in the other half of the trials in the control condition. Each dot represents one participant, the blue solid lines and the gray shadings are the fitted linear regression lines and their standard errors.

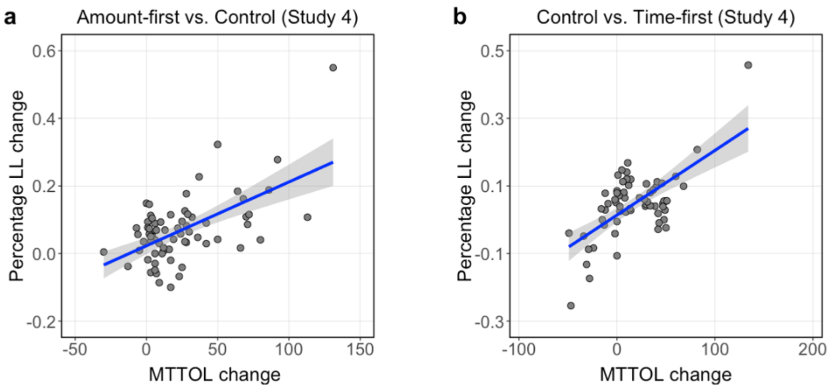

Supplementary Fig. 19. Correlations between the MTTOL change and the behavioral change across manipulation conditions in Study 4. (a) Amount-first vs. Control (two-sided Spearman correlation test, $\rho = 0.382$, $p = 0.001$, $S = 33851$); (b) Control vs. Time-first ($\rho = 0.406$, $p < 0.001$, $S = 27169$). Each dot represents one participant. The blue solid lines and the gray shadings are the fitted linear regression lines and their standard errors.

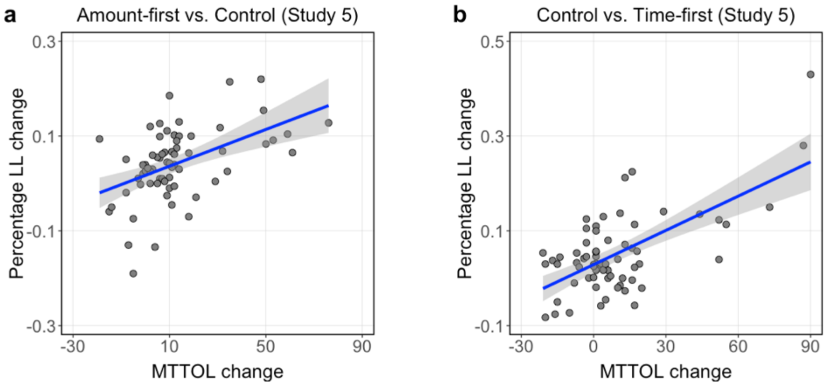

Supplementary Fig. 20. Correlations between the MTTOL change and the behavioral change across manipulation conditions in Study 5. (a) Amount-first vs. Control (two-sided Spearman correlation test, $\rho = 0.494$, $p < 0.001$, $S = 24264$); (b) Control vs. Time-first ($\rho = 0.330$, $p = 0.007$, $S = 32080$). Each dot represents one participant. The blue solid lines and the gray shadings are the fitted linear regression lines and their standard errors.

5.3 Mediation analysis for Studies 4 and 5

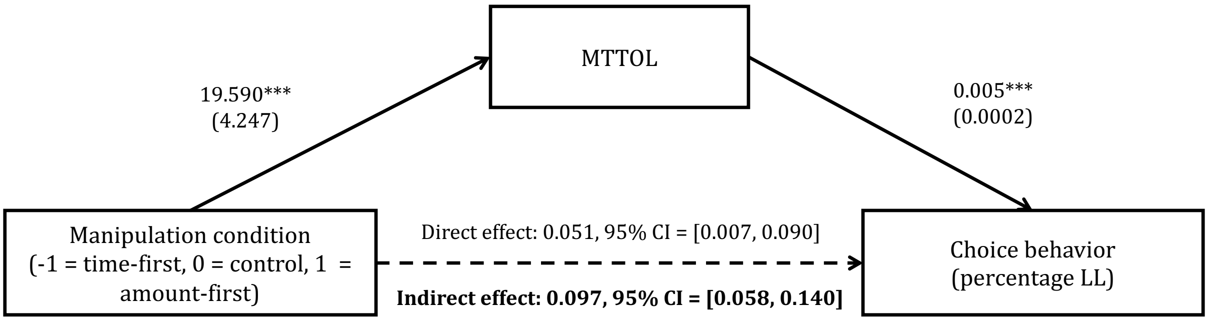

Supplementary Fig. 21. Mediation analysis for Study 4. The effect of manipulation on choice behavior was mediated via MTTOL. Model tests the indirect effect of manipulation condition (-1 = time-first, 0 = control, 1 = amount-first) on choice behavior (percentage of LL choices) through MTTOL by using a 95% confidence interval with 10,000 bootstrapped samples. The confidence interval for the indirect effect does not cross zero, indicating significant mediation. *$p<0.1$, **$p<0.05$, ***$p<0.01$.

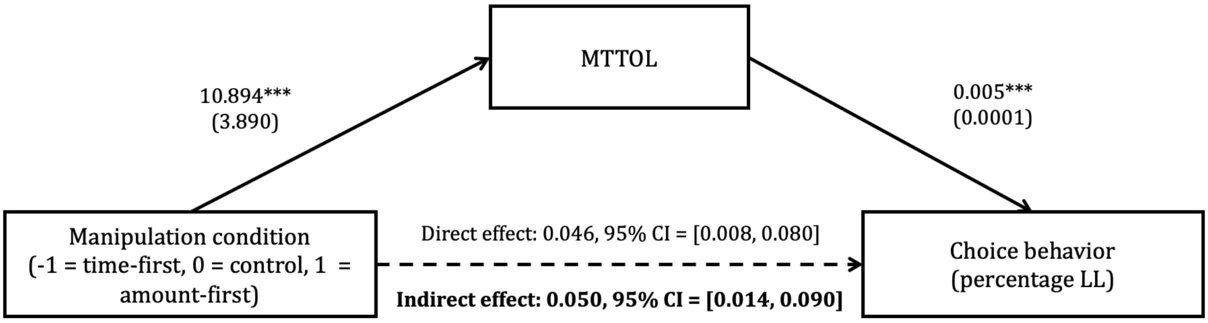

Supplementary Fig. 22. Mediation analysis for Study 5. The effect of manipulation on choice behavior was mediated via MTTOL. Model tests the indirect effect of manipulation condition (-1 = time-first, 0 = control, 1 = amount-first) on choice behavior (percentage of LL choices) through MTTOL by using a 95% confidence interval with 10,000 bootstrapped samples. The confidence interval for the indirect effect does not cross zero, indicating significant mediation. *$p<0.1$, **$p<0.05$, ***$p<0.01$.

Supplementary Note 6

Mouse-tracking analysis with/out trials shorter than 3 seconds in Study 4

In the manipulation conditions of Study 4, one attribute was displayed for 3 seconds and then the other attribute appeared (together with the first attribute). However, there were 17.2% of the trials in the time-first condition and 45.3% of the trials in the amount-first condition that were shorter than 3 seconds. That is, participants made decisions based on only one attribute in those trials. Here, we first excluded trials that were shorter than 3 seconds and then estimated the MTTOL based on the mouse-tracking data.

Supplementary Fig. 23 shows that the MTTOL change was correlated with the behavioral change across manipulations conditions (two-sided Spearman correlation tests, amount-first vs. control: $\rho = 0.758$, $p < 0.001$, $S = 8717$; control vs. time-first: $\rho = 0.421$, $p < 0.001$, $S = 26496$; amount-first vs. time-first: $\rho = 0.702$, $p < 0.001$, $S = 9189.1$).

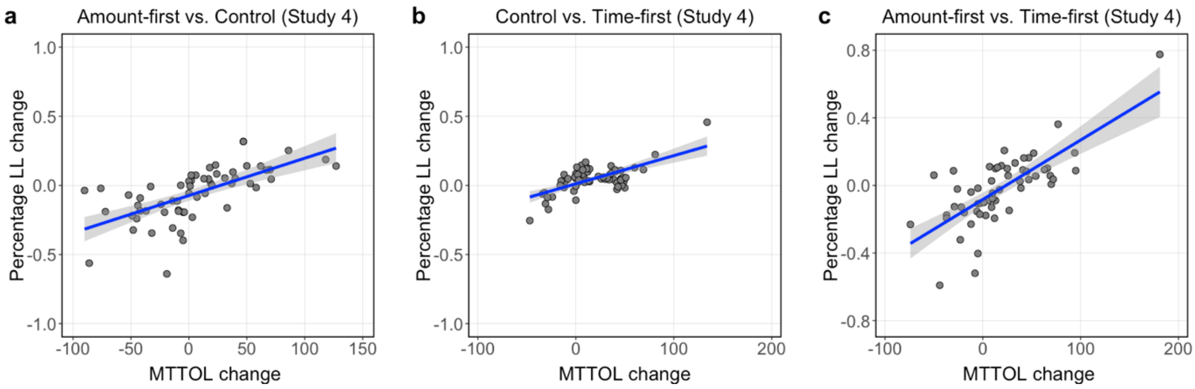

Supplementary Fig. 23. Correlations between the MTTOL change and the behavioral change across conditions in Study 4. (a) Amount-first vs. Control; (b) Control vs. Time-first; (c) Amount-first vs. Time-first. Trials shorter than 3 seconds were excluded when computing the MTTOL and the percentage of LL choices. Each dot represents one participant. The blue solid lines and the gray shadings are the fitted linear regression lines and their standard errors.

To check the robustness of the results above, in the following analysis we only excluded trials with extremely long RTs. Specifically, we eliminated trials when RTs were above the 0.75 quartile by more than 1.5 times the interquartile range. Then we estimated the MTTOL based on the mouse-tracking data in the remaining trials. Supplementary Fig. 24 shows that the MTTOL change was correlated with the behavioral change across manipulation conditions (two-sided Spearman correlation tests, amount-first vs. control: ρ = 0.381, p = 0.001, S = 33872; control vs. time-first: ρ = 0.310, p = 0.010, S = 37786) except amount-first versus time-first conditions (ρ = 0.213, p = 0.079, S = 43079).

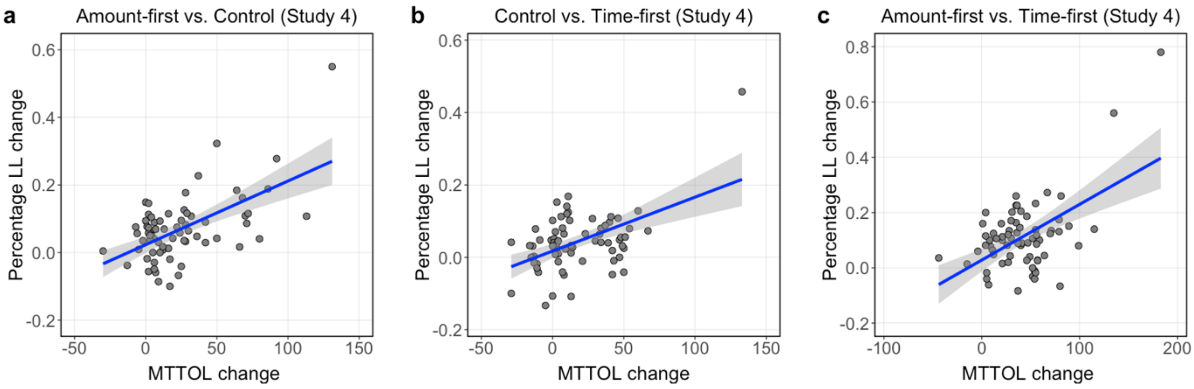

Supplementary Fig. 24. Correlations between the MTTOL change and the behavioral change across conditions in Study 4. (a) Amount-first vs. Control; (b) Control vs. Time-first; (c) Amount-first vs. Time-first. Trials with RTs that were above the 0.75 quartile by more than 1.5 times the interquartile range were excluded in this analysis. Each dot represents one participant. The blue solid lines and the gray shadings are the fitted linear regression lines and their standard errors.

Supplementary References

1. Wiecki T, Sofer I, Frank M. HDDM: Hierarchical Bayesian estimation of the Drift-Diffusion Model in Python. *Front Neuroinform* **7**, (2013).

2. Cramer JS. Predictive performance of the binary logit model in unbalanced samples. *J R Stat Soc Ser D* **48**, 85-94 (1999).

3. Clithero JA. Improving out-of-sample predictions using response times and a model of the decision process. *J Econ Behav Organ* **148**, 344-375 (2018).

4. Chen F, Krajbich I. Biased sequential sampling underlies the effects of time pressure and delay in social decision making. *Nat Commun* **9**, 3557 (2018).
