# Supplementary material for: Attribute latencies causally shape intertemporal decisions
Source: Nat Commun. 2024 Apr 5;15:2948. doi: 10.1038/s41467-024-46657-2 (PMC10997753; doi:10.1038/s41467-024-46657-2)
Supplement: Supplementary file 2 — Reporting Summary [file 41467_2024_46657_MOESM2_ESM.pdf]

nature portfolio

Corresponding author(s): Ian Krajbich

Last updated by author(s): Feb 8, 2024

Reporting Summary

Nature Portfolio wishes to improve the reproducibility of the work that we publish. This form provides structure for consistency and transparency in reporting. For further information on Nature Portfolio policies, see our Editorial Policies and the Editorial Policy Checklist.

Statistics

For all statistical analyses, confirm that the following items are present in the figure legend, table legend, main text, or Methods section.

n/a	Confirmed	
☐	☒	The exact sample size (*n*) for each experimental group/condition, given as a discrete number and unit of measurement
☐	☒	A statement on whether measurements were taken from distinct samples or whether the same sample was measured repeatedly
☐	☒	The statistical test(s) used AND whether they are one- or two-sided<br>*Only common tests should be described solely by name; describe more complex techniques in the Methods section.*
☐	☒	A description of all covariates tested
☒	☐	A description of any assumptions or corrections, such as tests of normality and adjustment for multiple comparisons
☐	☒	A full description of the statistical parameters including central tendency (e.g. means) or other basic estimates (e.g. regression coefficient) AND variation (e.g. standard deviation) or associated estimates of uncertainty (e.g. confidence intervals)
☐	☒	For null hypothesis testing, the test statistic (e.g. *F*, *t*, *r*) with confidence intervals, effect sizes, degrees of freedom and *P* value noted<br>*Give P values as exact values whenever suitable.*
☐	☒	For Bayesian analysis, information on the choice of priors and Markov chain Monte Carlo settings
☐	☒	For hierarchical and complex designs, identification of the appropriate level for tests and full reporting of outcomes
☐	☒	Estimates of effect sizes (e.g. Cohen's *d*, Pearson's *r*), indicating how they were calculated

Our web collection on statistics for biologists contains articles on many of the points above.

Software and code

Policy information about availability of computer code

Data collection	In Study 1, we collected data using MouseTracker (version 2.83) with a temporal resolution of 70Hz. In Studies 2 and 3 we programed the experiment using Python (version 3.8). In Studies 4 and 5, we programed the experiment using Python (version 3.8) and participants' mouse-trajectories were recorded with a temporal resolution of 70Hz.
Data analysis	R (version 4.1.3) was used to analyze the data. HDDM was used to estimate the vspDDM, i.e., a DDM with a varying starting point. And a hierarchical Bayesian toolbox available from https://github.com/galombardi/method_HtSSM_aDDM was used to fit the stDDM and other DDM variants. For all Bayesian modeling analyses, we used the default, uninformative priors specified by the respective R-packages. The code for the analyses presented in this article have been deposited to the Open Science Framework (https://osf.io/cy4gr/).

For manuscripts utilizing custom algorithms or software that are central to the research but not yet described in published literature, software must be made available to editors and reviewers. We strongly encourage code deposition in a community repository (e.g. GitHub). See the Nature Portfolio guidelines for submitting code & software for further information.

Data

Policy information about availability of data

All manuscripts must include a data availability statement. This statement should provide the following information, where applicable:

- Accession codes, unique identifiers, or web links for publicly available datasets
- A description of any restrictions on data availability
- For clinical datasets or third party data, please ensure that the statement adheres to our policy

The data generated in this study have been deposited to the Open Science Framework (https://osf.io/cy4gr/). Source data are provided with this paper.

Research involving human participants, their data, or biological material

Policy information about studies with human participants or human data. See also policy information about sex, gender (identity/presentation), and sexual orientation and race, ethnicity and racism.

Reporting on sex and gender	Sex and gender were not considered in the study design. We collected the gender information after they finished the task and they provided this information by self-reporting. Moreover, informed consent was obtained from all participants before the experiment.<br>70 out of 126 students were females in Study 1; 29 out of 49 students were females in Study 2; 22 of 43 students were females in Study 3; 43 out of 69 students were females in Study 4; 46 of 66 students were females in Study 5.
Reporting on race, ethnicity, or other socially relevant groupings	n/a
Population characteristics	In Study 1, there were 126 students, and 70 of them were females. The mean age was 22.7 years with a standard deviation of 2.4 years. In Study 2, there were 49 students, and 29 of them were females. The mean age was 22.9 years with a standard deviation of 2.7 years. In Study 3, there were 43 students, and 22 of them were females. The mean age was 23.3 years with a standard deviation of 2.6 years. In Study 4, there were 69 students, and 43 of them were females. The mean age was 22.56 years with a standard deviation of 2.6 years. In Study 5, there were 66 students, and 46 of them were females. The mean age was 21.62 years with a standard deviation of years.
Recruitment	Participants were recruited through an online platform at the university. Students in this university could browse and sign up for studies.
Ethics oversight	The Institutional Review Board of the Neuro-Management Lab at Zhejiang University approved the study protocol.

Note that full information on the approval of the study protocol must also be provided in the manuscript.

Field-specific reporting

Please select the one below that is the best fit for your research. If you are not sure, read the appropriate sections before making your selection.

☐ Life sciences ☒ Behavioural & social sciences ☐ Ecological, evolutionary & environmental sciences

For a reference copy of the document with all sections, see nature.com/documents/nr-reporting-summary-flat.pdf

Behavioural & social sciences study design

All studies must disclose on these points even when the disclosure is negative.

Study description	This study is a behavioral and mouse-tracking study. We collected participants' mouse-trajectory data, choice data and response time data using five experiments. The data are quantitative.
Research sample	126 students (70 females, mean age = 22.7 years, s.d. = 2.4 years) from Zhejiang University, China participated in Study 1, 49 students (29 females, mean age = 22.9 years, s.d. = 2.7 years) participated in Study 2, 43 students (22 females, mean age = 23.3 years, s.d. = 2.6 years) participated in Study 3, 69 students (43 females, mean age = 22.56 years, s.d. = 2.6 years) participated in Study 4, and 66 students (46 females, mean age = 21.62 years, s.d. = 2.1 years) participated in Study 5. The sample is representative. We chose this study sample similar to previous studies (Chen & Krajbich, 2018, Chen et al., 2023, Fisher, 2021, Sullivan & Huettel, 2021).
Sampling strategy	No statistical methods were used to predetermine sample sizes. But our sample size is larger than those reported in previous publications (Chen & Krajbich, 2018, Chen et al., 2023, Fisher, 2021, Sullivan & Huettel, 2021). We aimed to collect at least 120 participants for Study 1, and at least 40 participants each for Studies 2-5.
Data collection	In Study 1, we tracked the mouse-trajectories of each decision using MouseTracker with a temporal resolution of 70Hz. In Studies 2 and 3, we programed the experiment using Python and did not record the mouse-trajectories. In Studies 4 and 5, we programed the experiment using Python and participants' mouse-trajectories were recorded with a temporal resolution of 70Hz. The researcher was

	not blind to experimental condition since different conditions in each study required different instructions. During the experiment, the researcher sit in the experimenter room. No one was present besides the participants and the researcher.
Timing	Data collection for Study 1 took place from June 2019 to June 2020 (interrupted by the COVID-19). Data collection for Study 2 took place in Apr. 2021, data collection for Study 3 took place in May 2021, data collection for Study 4 took place in Jan. 2022, and data collection for Study 5 took place in Mar. 2022.
Data exclusions	No data were excluded from the analyses.
Non-participation	No participants dropped out/declined participation in this study.
Randomization	In Study 1, all participants made decisions in each of the three time-manipulation conditions. In Studies 2-5, all participants made decisions in each of the three information-display conditions. We randomized the order of the games within each condition, as well as the SS and LL options on the left and right sides of the display.

Reporting for specific materials, systems and methods

We require information from authors about some types of materials, experimental systems and methods used in many studies. Here, indicate whether each material, system or method listed is relevant to your study. If you are not sure if a list item applies to your research, read the appropriate section before selecting a response.

Materials & experimental systems

n/a	Involved in the study
☒	☐ Antibodies
☒	☐ Eukaryotic cell lines
☒	☐ Palaeontology and archaeology
☒	☐ Animals and other organisms
☒	☐ Clinical data
☒	☐ Dual use research of concern
☒	☐ Plants

Methods

n/a	Involved in the study
☒	☐ ChIP-seq
☒	☐ Flow cytometry
☒	☐ MRI-based neuroimaging

Plants

Seed stocks	n/a
Novel plant genotypes	n/a
Authentication	n/a
